# Solvent- and Catalyst-free Synthesis, Hybridization and Characterization of Biobased Nonisocyanate Polyurethane (NIPU)

**DOI:** 10.3390/polym11061026

**Published:** 2019-06-10

**Authors:** Xin He, Xiaoling Xu, Qian Wan, Guangxu Bo, Yunjun Yan

**Affiliations:** Key Laboratory of Molecular Biophysics of the Ministry of Education, College of Life Science and Technology, Huazhong University of Science and Technology, Wuhan 430074, China; n785888@163.com (X.H.); xuxiaoling@hust.edu.cn (X.X.); m201671694@hust.edu.cn (Q.W.); m201671171@hust.edu.cn (G.B.)

**Keywords:** hybrid nonisocyanate polyurethane, solvent- and catalyst-free, dimer acid, melt condensation

## Abstract

Nonisocyanate polyurethane (NIPU) is a research hotspot in polyurethane applications because it does not use phosgene. Herein, a novel method of solvent- and catalyst-free synthesis of a hybrid nonisocyanate polyurethane (HNIPU) is proposed. First, four diamines were used to react with ethylene carbonate to obtain four bis(hydroxyethyloxycarbonylamino)alkane (BHA). Then, BHA reacted with dimer acid under condensation in the melt to prepare four nonisocynate polyurethane prepolymers. Further, the HNIPUs were obtained by crosslinking prepolymers and resin epoxy and cured with the program temperature rise. In addition, four amines and two resin epoxies were employed to study the effects and regularity of HNIPUs. According to the results from thermal and dynamic mechanical analyses, those HNIPUs showed a high degree of thermal stability, and the highest 5% weight loss reached about 350 °C. More importantly, the utilization of these green raw materials accords with the concept of sustainable development. Further, the synthetic method and HNIPUs don’t need isocyanates, catalysts, or solvents.

## 1. Introduction

Owing to their excellent surface protection, chemical and abrasion resistance, mechanical properties, high elasticity, and good biocompatibility, polyurethanes (PUs) have become one of the most versatile polymers, with wide application in many fields, such as furnishing, cars, clothing, shoes, elastomers, coatings, walls, and roofing insulation [1,2,3,4,5,6,7,8,9,10]. However, considering the potential harm to the environment and human health by cyanate and phosgene (raw materials for isocyanate), developing non-isocyanate polyurethanes (NIPUs) is a question that needs an immediate answer [9,11,12].

Today, the sustainable development of raw materials is becoming imperative for human beings, as a result of the lack of fossil fuels [13]. The current challenge in the polyurethane industry is to switch from petro-based polyurethane to bio-based PUs or NIPUs [11,14]. In traditional bio-based PUs, vegetable oil was one of the major raw materials because of its convenient designability of double bonds and ester bonds [15,16]. Kong et al. utilized canola, sunflower, and camelina oil to synthesize bio-based polyols, which were the starting materials for the production of PU coating [17]. Wu et al. applied natural *Sapium sebiferum* oil to produce polyol, the hydroxyl value of which reached to 211 mgKOH/g. Finally, the synthesized PU featured good thermal and mechanical properties [18]. Campanella et al. synthesized bio-based polyurethane foams from soybean oil and proved it was feasible [19]. Kirpluks et al. obtained high functional polyols from rapeseed oil, aiming to prepare rigid PU foam thermal insulation material [20]. Vanags et al. used epoxidized tall oil fatty acids to synthesize polyol, which had a high OH value (reaching to 527 ± 2 mg KOH/g) for polyurethane application [21]. Furthermore, dimer acid, derived from fatty acids, was a renewable resource and can be an alternative for PU [22,23,24]. Although dimer acid is a value-added product, a few conversion technologies have been proposed by researchers. Yao et al. used dimer acid and ethylene glycol to synthesize polyester polyol and then obtained PU via a reaction with isophorone diisocyanate [25]. NIPU can also use these sustainable resources as the main raw materials. Specifically, bio-based cyclic carbonates have been an important research topic, including the epoxidation of vegetable oils, terpenes, or vanillin derivates, or by the glycidylization of bio-polyols followed by carbonation with carbon dioxide [26,27].

**Scheme 1:**
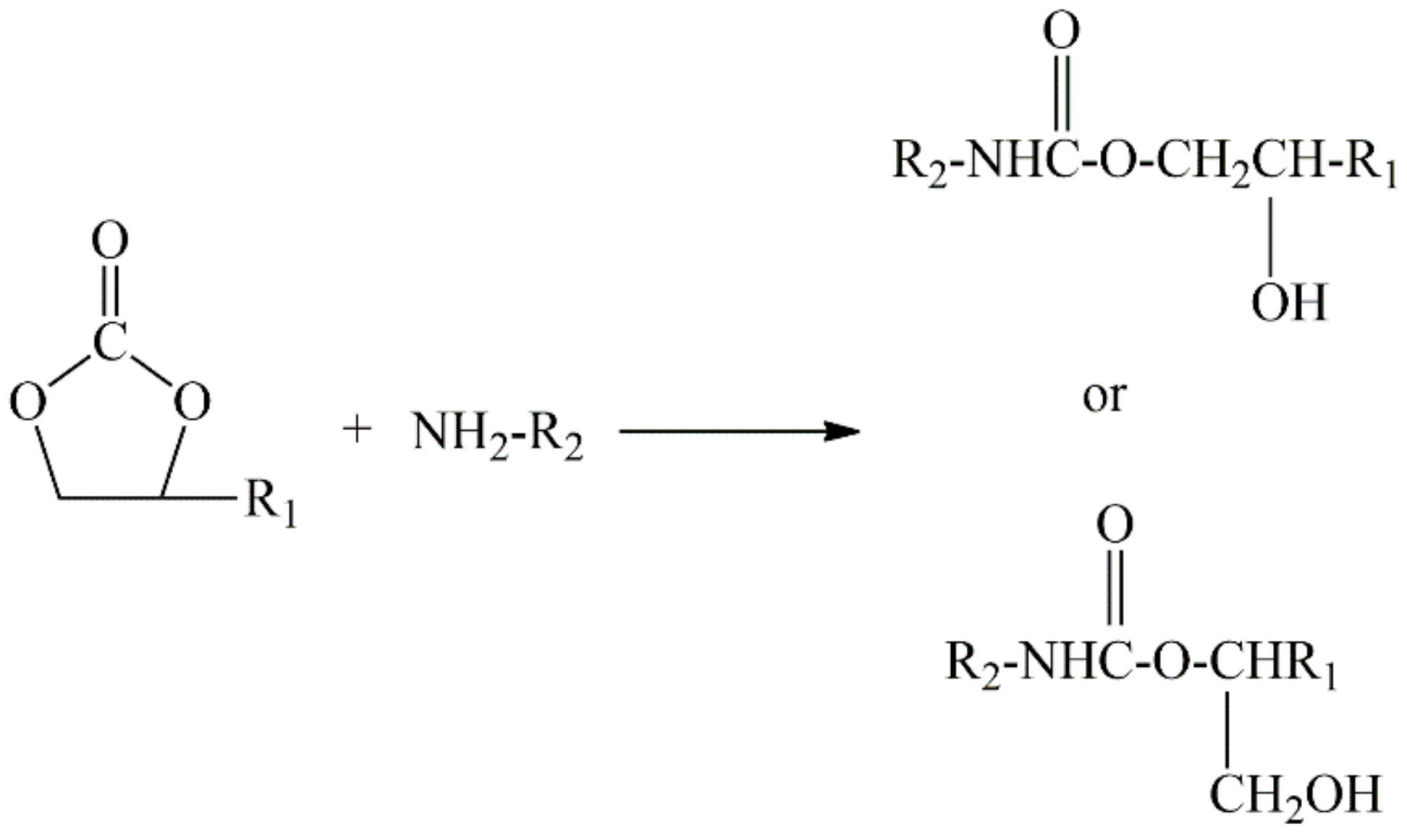
Ring-opening synthesis of non-isocyanate polyurethanes (NIPU).

Today, many studies of NIPU have been reported, and most of the synthetic methods (as shown in Scheme 1) were ring-opening polymerizations of cyclic carbonates via diamines or multi-amines [28,29]. Therefore, the synthesis of cyclic carbonate oligomers/polymers has represented the majority of research on NIPU preparation [30,31]. However, few studies have been industrialized because of the raw material cost, safety, construction difficulty, and so on. Naturally, searching alternative methods for NIPU preparation has also become an issue worth thoroughly investigating. Among them, the carbamate monomer (bis-hydroxycarbamate), synthesized by a cyclocarbonate and a diamine, is an appropriate option. This way, the carbamate monomer can react with polyol or polyacid to produce NIPU by a polycondensation reaction. Rokicki et al. used bis-hydroxycarbamate, synthesized by ethylene carbonate and diamine (1, 4-diaminobutane or 1, 6-diaminohexane), to react with diol (1, 6-hexanediol or 1, 10-decanediol), and obtained several NIPUs [32]. However, the disadvantages of the synthesized NIPUs by this route cannot be ignored, especially the low glass transition temperature, which is a consequence of the low average molecular weight [3,4,33]. To solve this problem, appropriate catalysts, chain extenders, and crosslinking agents are worthwhile choices.

Therefore, in this study, for the purposes of renewable and sustainable development, dimer acid (DA) was selected as the biomass with a flexible chain and low glass transition temperature [34,35]. Ethylene carbonate (EC) was chosen as the key component of the urethane group because it can be synthesized by ethylene oxide and carbon dioxide and is also a green raw material [36]. Furthermore, as is known, fixing carbon dioxide is one of the potential ways to resolve the greenhouse gas problem [37,38]. First, a series of bis(hydroxyethyloxycarbonylamino)alkanes (BHAs) were synthesized from several amines and EC, and then the BHAs reacted with the DA to produce an NIPU-prepolymer by melt polycondensation. To solve the problem of low average molecular weight, epoxy resins were employed to crosslink the NIPU-prepolymer with the secondary amine. For characterization of those products, finding out the regularity of the epoxy value and secondary amine for hybrid nonisocyanate polyurethane (HNIPU) and exploring whether them have potential for coating applications, several instruments, including Fourier transform infrared spectroscopy (FTIR), nuclear magnetic resonance (NMR), Fourier transform mass spectrometer (FTMS), Gel permeation chromatography (GPC), thermogravimetric analysis (TGA), differential scanning calorimetry (DSC), dynamic mechanical analysis (DMA), field emission scanning electron microscopy (FSEM), X-ray diffraction (XRD), Atomic force microscope (AFM), water absorption, and swelling, were used in this study.

## 2. Materials and Methods 

### 2.1. Materials

Ethylene carbonate (EC, 99%), ethanediamine (EDA, 99%), diethylenetriamine (DETA, 100%), triethylenetetramine (TETA, 100%) and tetraethylenepentamine (TEPA, 90%) were purchased from Sinopharm Chemical Reagent Ltd Co. (Shanghai, China). All the materials were analytical reagents and used without any extra processing. Dimer acid (DA, 98%, 9-[(Z)-non-3-enyl]-10-octylnonadecanedioic acid) was bought from Bangcheng Chemical Ltd. Bisphenol-A epoxy resin E-44 (mean epoxy value: 0.44) and Bisphenol-A epoxy resin E-51 (mean epoxy value: 0.51) were obtained from Yueyang Petrochemical Ltd Co. (Yueyang, China).

### 2.2. Synthesis of BHAs

**Scheme 2:**
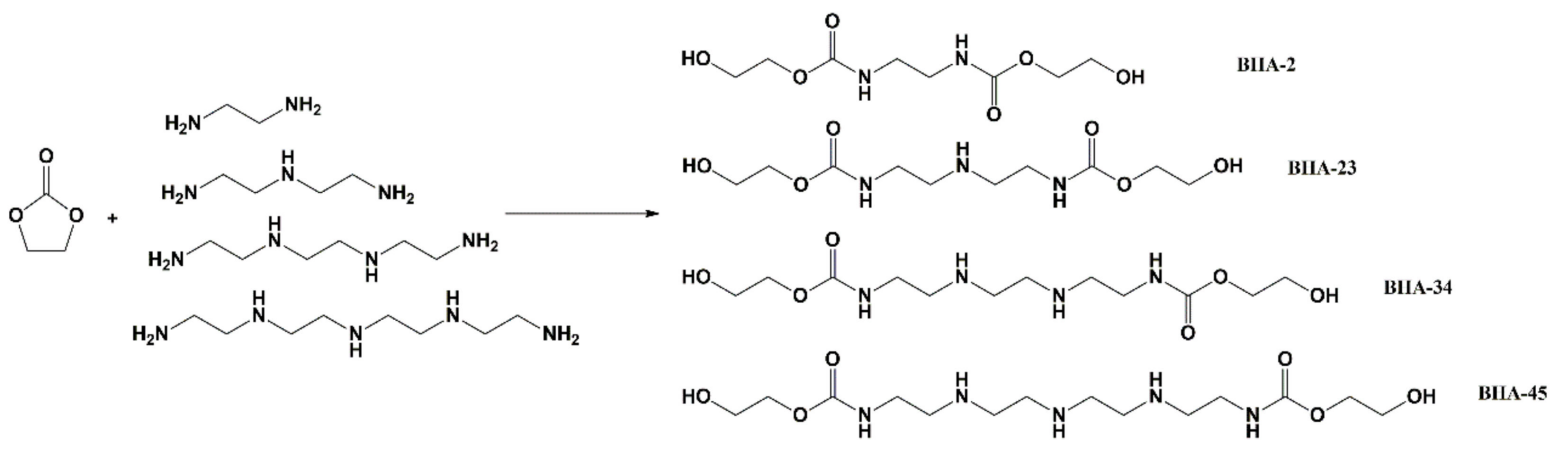
Synthesis of bis(hydroxyethyloxycarbonylamino)alkanes (BHAs).

BHAs were synthesized from EC and four amines (EDA, DETA, TETA, and TEPA) by a ring opening reaction (seen in Scheme 2). A 100.0 g amount of EC was weighed into a 500 mL three-necked round-bottomed flask, and equimolar amine (EDA:34.1 g; DETA: 58.0 g; TETA: 82.2 g; TEPA: 118.2 g) was put into a constant pressure separation funnel. Then, both were kept in a 60 ℃ oil bath with magnetic stirring, and the amine was added dropwise. The mixture was stirred continuously until no bubbles appeared. Then the temperature was increased to 100 ℃ and kept for four hours. After cooling to room temperature, the sample was washed three times with acetonitrile and the solution was dumped. Subsequently, the products were dried under a 60 ℃ vacuum drying oven for a day. On the basis of four amines, the derived BHAs were named BHA-2 (obtained 127.4 g, yield 95% ), BHA-23 (obtained 148.5 g, yield 94% ), BHA-34 (obtained 174.9 g, yield 96%), and BHA-45 (obtained 207.3, yield 95%). The detailed raw material mass and results of the BHAs are listed in Table 1. BHA-2, BHA-23, and BHA-34 were a light-yellow powder with a little viscidity. BHA-45 was a yellow paste with some viscidity.

### 2.3. Synthesis of NIPU Prepolymers

**Scheme 3:**
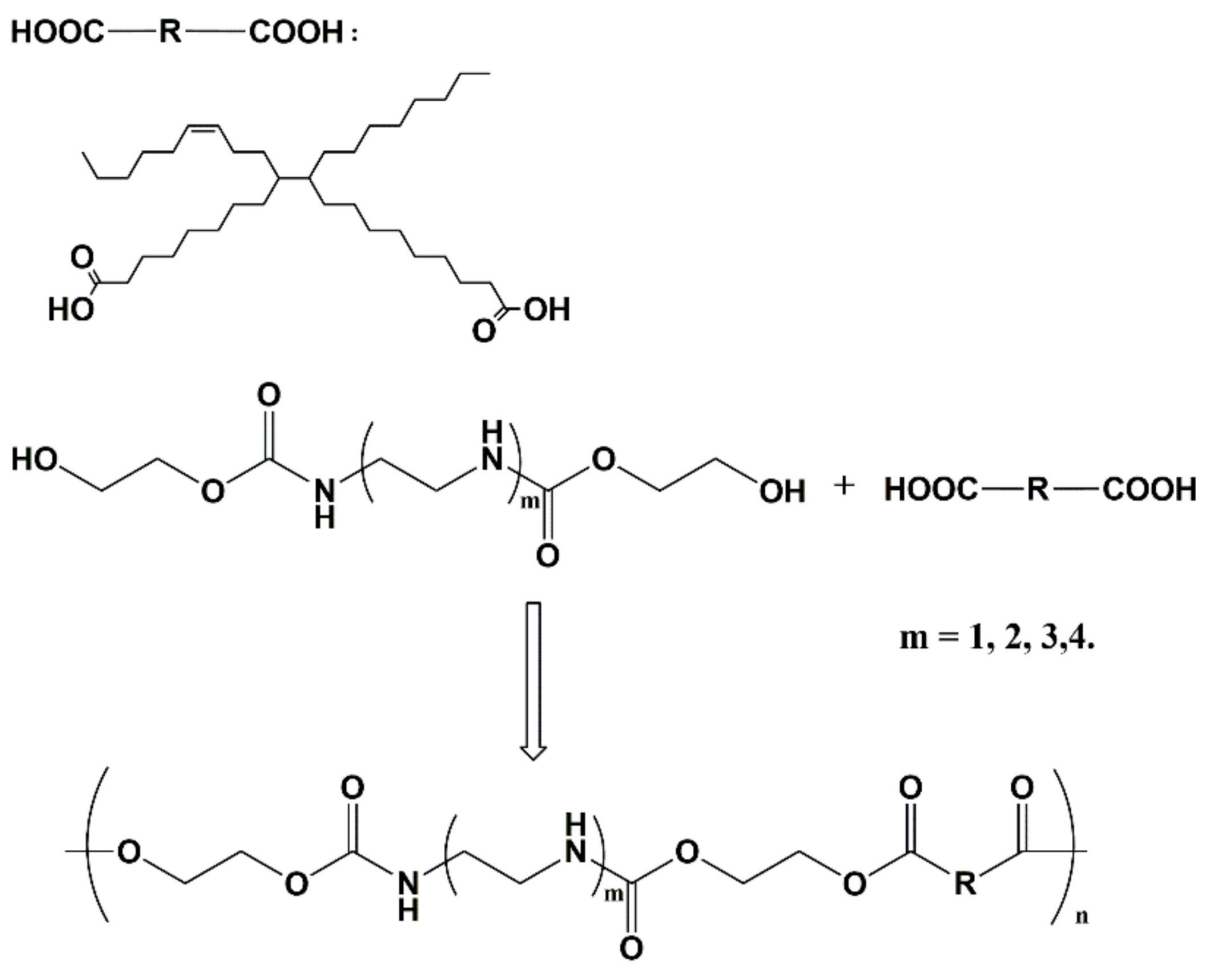
Synthesis of the NIPU prepolymer.

As Scheme 3 shows, the reaction is an esterification reaction. After mixing 10.0 g BHA with a little excess of dimer acid (reactant molar ratio = 1:1.05), the reaction was set in a 250 mL three-necked round-bottomed flask with magnetic stirring, and reacted at 180 °C for 4 h under nitrogen blowing. Then, the nitrogen pipe was withdrawn and the reaction was continued with a reduced pressure aimed at removing the water to promote the esterification reaction. Finally, the samples were poured into a beaker and dried at 60 °C in a vacuum drying oven. Corresponding to the different BHAs, the NIPU prepolymers were named NIPU-2, NIPU-23, NIPU-34, and NIPU-45. The detailed raw material mass and results of the NIPUs are listed in Table 2. NIPU-2, NIPU-23, and NIPU-34 were a brown paste with some viscidity. NIPU-45 was a brown colloid with little viscidity.

### 2.4. Synthesis of HNIPUs

**Scheme 4:**
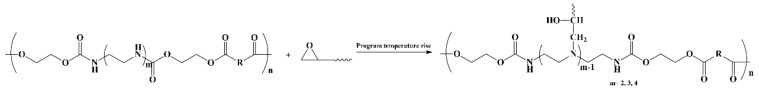
Synthesis of hybrid nonisocyanate polyurethanes (HNIPUs).

To increase the crosslinking degree, epoxy resins were employed to react with the secondary amines, which were produced by the polyethyene polyamine. Therefore, the NIPU-2 was excluded from this step because of the lack of a secondary amine. First, 2.0 g NIPU prepolymer were taken into a flask, and kept in a 60 °C oil bath with magnetic stirring. When the samples reverted to the liquid stage, 1.4 g of epoxy resin was added; then, the mixture was stirred and the bubble was eliminated by vacuum. When the mixture turned to a homogeneous phase it was poured into a teflon mold and kept on a 60 °C vacuum drying oven for 12 h. Subsequently, the temperature was changed to 90 °C and the HNIPUs were cured for 4 h. After naturally cooling to room temperature, bulk specimens were obtained (3.4 g, yield 100%, Scheme 4), packed, and kept for characterization. The detailed raw material mass and results of HNIPUs are listed in Table 3. HNIPU-2344 and HNIPU-2351 were a light-yellow paste with some viscidity. HNIPU-3444 (ρ = 1.02 g/cm^3^), HNIPU-3451 (ρ = 1.26 g/cm^3^), HNIPU-4544 (ρ = 1.01 g/cm^3^), and HNIPU-4551 (ρ = 1.35 g/cm^3^) were yellow elastic with non-viscidity. There were no bubbles in all these samples.

### 2.5. Characterization

The ^1^H nuclear magnetic resonance (NMR) spectra of samples were determined by a Bruker AV600 MHz NMR spectrometer (Bruker, Karlsruhe, Germany). BHA-2 was dissolved by dimethylsulfoxide (DMSO) and the other BHAs were dissolved by Deuteroxide (Tetramethylsilane as an internal standard), and all mixtures were injected into 5 mm diameter NMR sample tubes. All of the ^1^H-NMR spectra were measured at room temperature.

A Bruker Vertex 70 Fourier transform infrared spectroscopy (FTIR) spectrometer (Bruker, Karlsruhe, Germany), equipped with an attenuated total reflection (ATR) accessary, was employed to test the FTIR spectra. Liquid samples were smeared on a KBr tablet, and solid block samples were test by ATR accessary. All samples were scanned from 4000 to 40 cm^−1^ with a resolution of 4 cm^−1^ with 128 scans at room temperature.

A Solari 7.0T high resolution Fourier transform mass spectrometer (FTMS) (Bruker, Karlsruhe, Germany) was employed to determine the mass-to-charge ratios by electrospray ionization (ESI). The BHAs were dissolved into water and injected for characterization. 

The weight average molecular weight (*M*w) and polymer dispersity index (PDI = *M*w/*M*n, *M*n was number average molecular weight) were measured by using an SSI Series 1500 Gel permeation chromatography (GPC) instrument (SSI, USA), which was equipped with a refractive index detector and Shodex KF-802.5 chromatogram column. The temperature was set to 40 °C, and tetrahydrofuran (THF) was used as eluent at a flow rate of 1.0 mL/min.

A Pyris 1 thermogravimetric analysis (TGA) instrument (Perkin-Elmer, Waltham, MA, USA) was employed to study the thermostability of samples and performed from room temperature to 600 °C with a heating rate of 10 °C /min in N_2_ atmosphere.

The glass transition temperature (*T*_g_) was tested by using a Perkin-Elmer Diamond DSC instrument (Perkin-Elmer, Waltham, MA, USA). The testing conditions included increasing the temperature to 150 °C at a rate of 30 °C /min and keeping it at 150 °C for 2 min to eliminate the thermal history of a sample; then, decreasing the temperature to −50 °C at a rate of 5 °C/min and keeping it at –50 °C for 2 min. Finally, the temperature was increased from −50 to 150 °C at a rate of 10 °C/min. The last heating curve was recorded for analysis. During the test, the N_2_ atmosphere was kept stable.

Dynamic mechanical analysis (DMA) was performed on a Perkin-Elmer Diamond DMA instrument (Perkin-Elmer, Waltham, MA, USA). All HNIPUs were cut into a 1 mm × 10 mm × 30 mm rectangular block and heated from −100 °C to 150 °C at a heating rate of 3 °C /min under a N_2_ atmosphere with a single cantilever mode in the testing process. 

A sirion 200 field emission scanning electron microscopy (FSEM) instrument (FEI, Eindhoven, Netherlands) was employed to observe the morphology of the HNIPUs. All samples were freeze-fractured after a short immersing time in liquid nitrogen and sputter coated with a Au layer. 

X-ray diffraction (XRD) was utilized to confirm the crystallization behavior of the samples and characterized by using a x’ pert3 powder X-ray diffractometer. A 10 mm × 10 mm flakelet was placed on the instrument with a 2θ range from 5° to 50° at 5 °/min.

An atomic force microscope (AFM) analysis was performed using an SPM9700 AFM instrument (Shimadzu, Kyoto, Japan) with a tapping mode. Samples were cut into flat pieces, and the surface was cleared by absolute ethanol. Then, the samples were dried at 60 °C for 24 h.

The water absorption of all HNIPUs was tested from the weight change of a film sample before and after the immersion in deionized water at room temperature over time. The samples of the HNIPUs were taken out and immediately wiped with filter paper and accurately weighed. The following equation was calculation for water absorption:

(1) $$\mathrm{Water}\;\mathrm{absorption}\left(\%\right)=\frac{W_{a}-\mathrm{Wo}}{\mathrm{Wo}}\times 100$$

where *W*_a_ and *W*_o_ are the weight of samples after and before immersion in deionized water, respectively.

A swelling test was determined by the mass change of a film sample under immersion in dimethylsulfoxide (DMSO) at room temperature. After taking out the sample, a filter paper was used to dry the residual DMSO on the surface and accurately weigh the mass. The swelling was calculated by the following equation:

(2) $$\mathrm{Swelling}\left(\%\right)=\frac{W_{t}-\mathrm{Wo}}{\mathrm{Wo}}\times 100$$

where *W*_t_ and *W*_o_ are after and before immersion in DMSO, respectively.

## 3. Results and Discussion

### 3.1. Synthesis of BHAs

**Figure 1:**
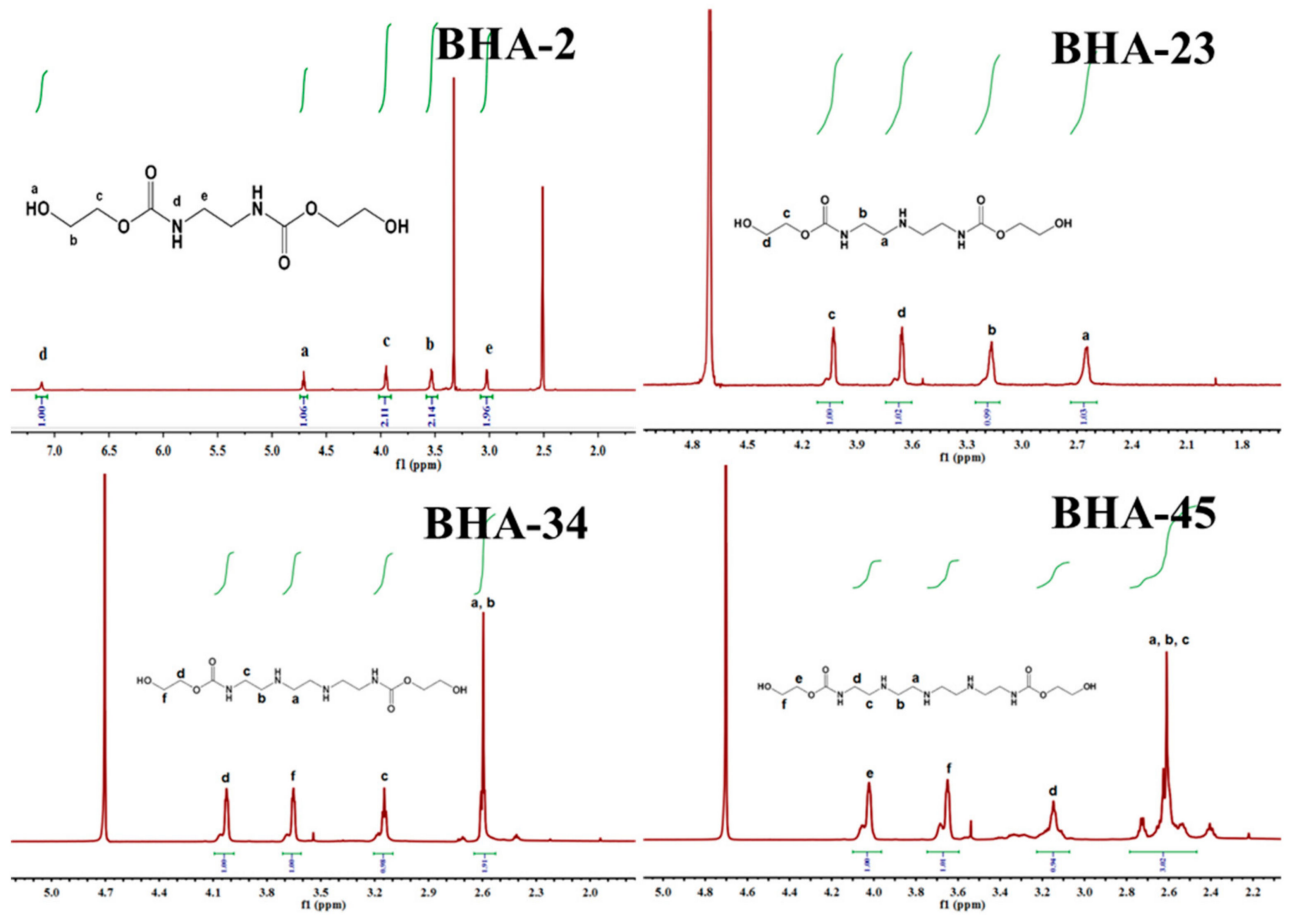
^1^H nuclear magnetic resonance (NMR) spectra of four BHAs. (**BHA-2**) ^1^H NMR (DMSO-*d*_6_): δ (ppm) = 3.03 (–C(O)–NH–CH_2_–), 7.12 (–C(O)–NH–CH_2_–), 3.94 (HO–CH_2_–CH_2_–O–), 3.54 (HO–CH_2_–CH_2_–O–), 4.70 (HO–CH_2_–CH_2_–O–). (**BHA-23**) ^1^H NMR (D_2_O): δ (ppm) = 2.64 (–C(O) –NH–CH_2_–CH_2_–NH–), 3.17 (–C(O)–NH–CH_2_–CH_2_–NH–), 3.65 (HO–CH_2_–CH_2_–O–), 4.03 (HO–CH_2_–CH_2_–O–). (**BHA-34**) ^1^H NMR (D_2_O): δ (ppm) = 2.59–2.61 (–C(O)–NH–CH_2_–CH_2_–NH–CH_2_–), 3.16 (–C(O)–NH–CH_2_–CH_2_–NH–CH_2_–), 3.65 (HO–CH_2_–CH_2_–O–), 4.02 (HO–CH_2_–CH_2_–O–). (**BHA-45**) ^1^H NMR (D_2_O): δ (ppm) = 2.46–2.73 (–C(O)–NH–CH_2_–CH_2_–NH–CH_2_–CH_2_), 3.15 (–C(O)–NH–CH_2_–CH_2_–NH–CH_2_–CH_2_), 3.66 (HO–CH_2_–CH_2_–O–), 4.02 (HO–CH_2_–CH_2_–O–).

Few reports have described the synthesis of BHAs, especially utilizing polyene polyamine. In this section, four BHAs were first obtained via ring opening reaction and confirmed by NMR, FTIR, and FTMS. In Figure 1, because of the different solubility of BHAs, the Figure shows that the dissimilar solvent peak: δ (ppm) = 2.50 (DMSO-d6, solvent peak) and 3.30 (H_2_O, water peak) belonged to Figure 1a; δ (ppm) = 4.70 (D_2_O, solvent peak) belonged to Figure 1b–d. Figure 1 shows the chemical shift of different hydrogen atom, and confirmed successful synthesis of the four BHAs. 

**Figure 2:**
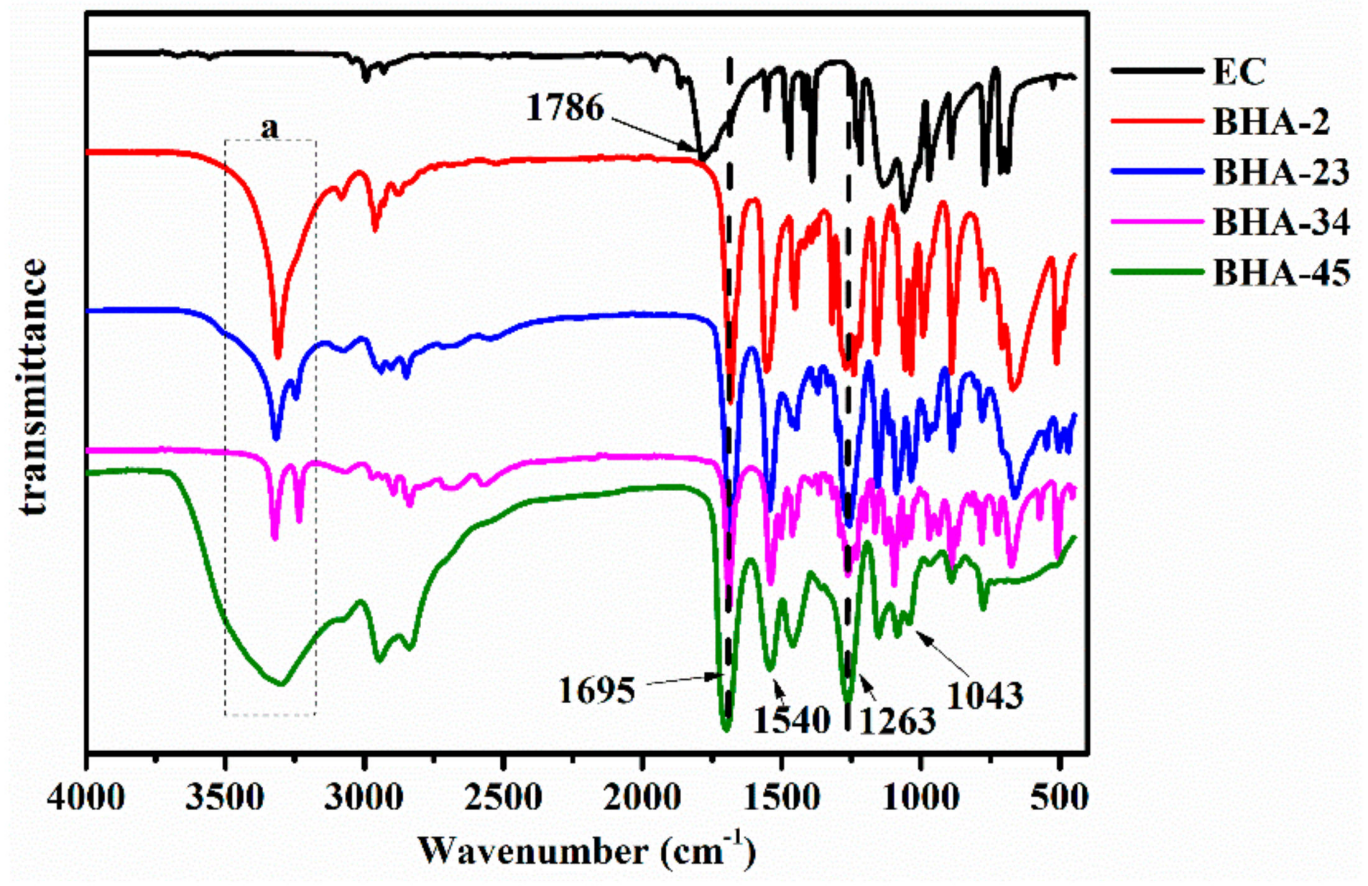
Fourier transform infrared spectroscopy (FTIR) spectra of four BHAs.

Figure 2 shows the FTIR spectra of four BHAs. From the dotted box of **a**, as the interaction of –OH (*v*, 3400 cm^−1^) and –NH– (*v*, 3300 cm^−1^), the stretching vibration changed with an increasing number of secondary amines, especially BHA-45. The rest of the characteristic peaks are as follows: 2942 cm^−1^ (*v*_as_, –CH_2_–), 2836 cm^−1^ (*v*, –CH_2_–), 1695 cm^−1^ (*v*, C=O), 1540 cm^−1^ (*δ*, N–H), 1043 cm^−1^ (*v*, C–OH). The band characteristic for the urethane group was proven to successfully form via 1695 cm^−1^ (*v*, C=O) and 1540 cm^−1^ (*δ*, N–H). Further, by contrasting with EC, the vibration peak at 1786 cm^−1^ for BHAs disappeared because of the ring opening of EC.

Figures S1–S4 show the FTMS spectra of four BHAs, respectively. The corresponding relative molecular mass of BHAs are as follows: BHA-2, 236.10 g/mol; BHA-23, 279.14 g/mol; BHA-34, 322.19 g/mol; BHA-45, 365.23 g/mol. In contrast, the FTMS showed the [M+H], and confirmed the synthesis and purity of the BHAs.

**Figure 3:**
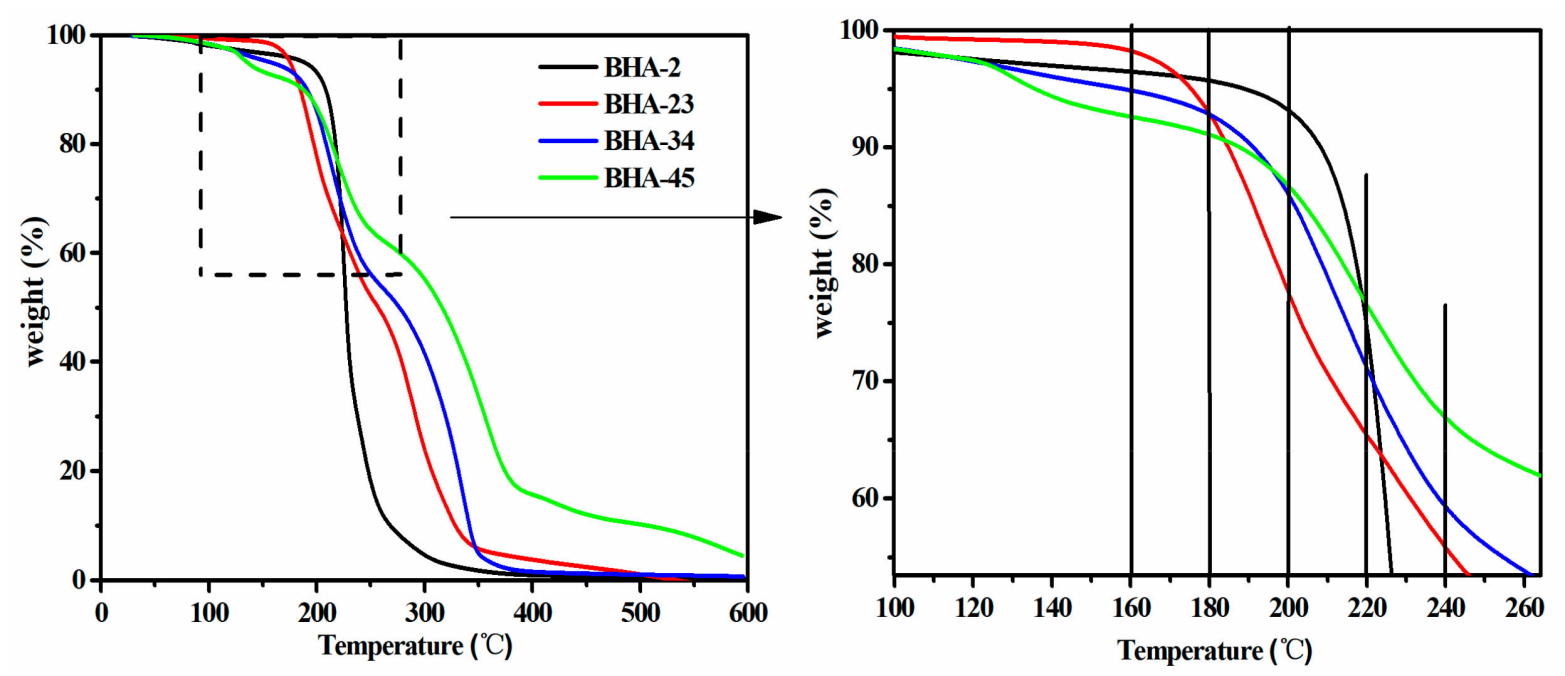
Thermogravimetric analysis (TGA) curves of four BHAs.

Figure 3 provides the TGA curves of the BHAs. When the temperature exceeded 180 °C, there was a sharp decrease in the weight loss of the four BHAs. Thus, In consideration of the decomposition and reactivity of the BHAs, 180 °C would be an appropriate temperature for the synthesis of a NIPU-prepolymer. 

### 3.2. Synthesis of NIPU-prepolymers

**Figure 4:**
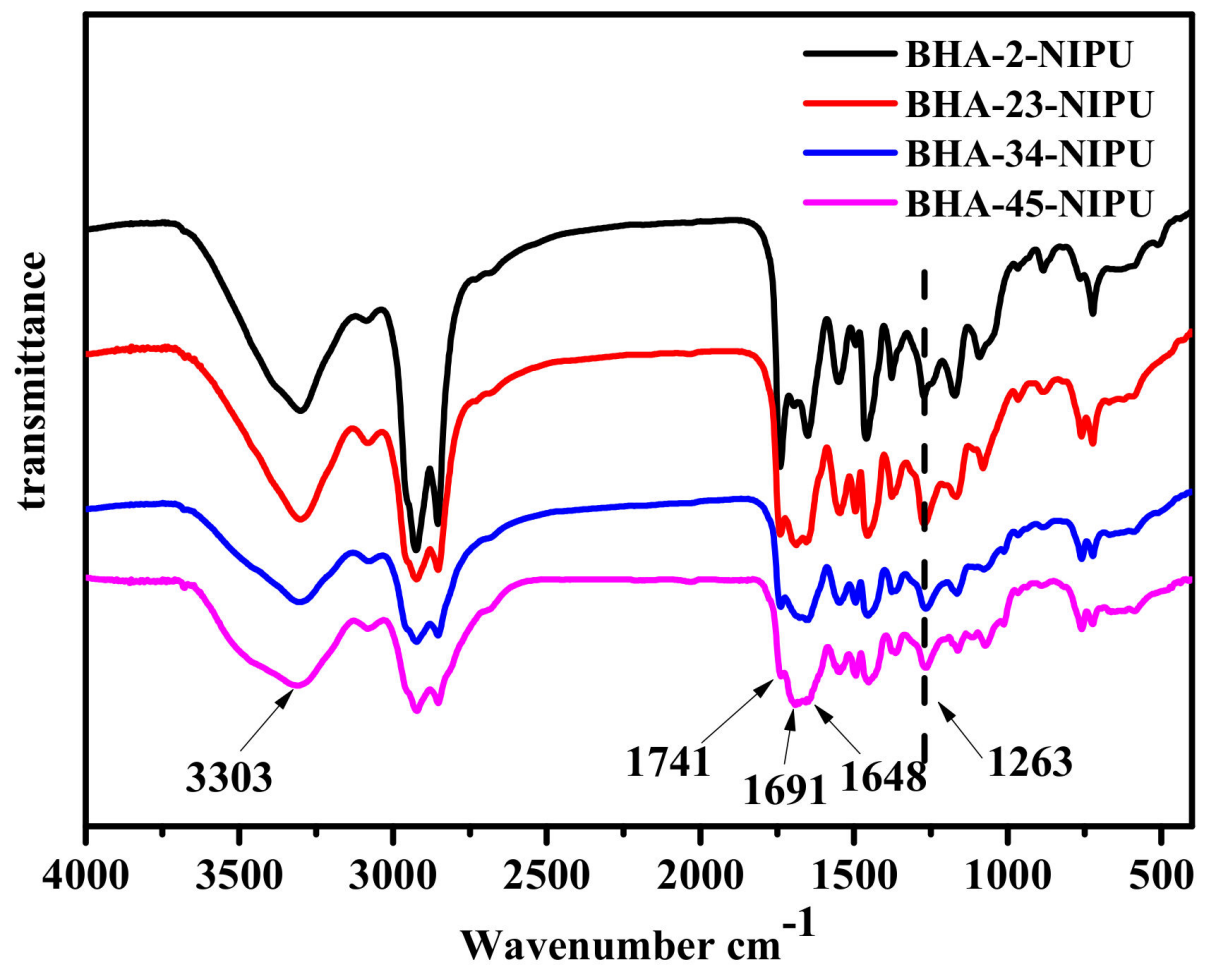
FTIR spectra of four NIPU-prepolymers.

Figure 4 shows the FTIR spectra of different NIPU-prepolymers. The main vibration peaks were indicated by arrows: 3303 cm^−1^ (*v*, –NH–), 1741 cm^−1^ (*v*, C=O, from carboxyl groups), 1691 cm^−1^ (*v*, C=O, from urethane groups), 1648 cm^−1^ (*v*, C=O, from ester groups). Compared with Figure 2, a new vibration peak appears at 1648 cm^−1^ in Figure 4 and confirmed the formation of ester groups. The vibration peak at 3303 cm^−1^ increased along with the increase of the secondary amine. The vibration peak at 1741 cm^−1^ proved that the prepolymer was blocked with dimer acid.

**Figure 5:**
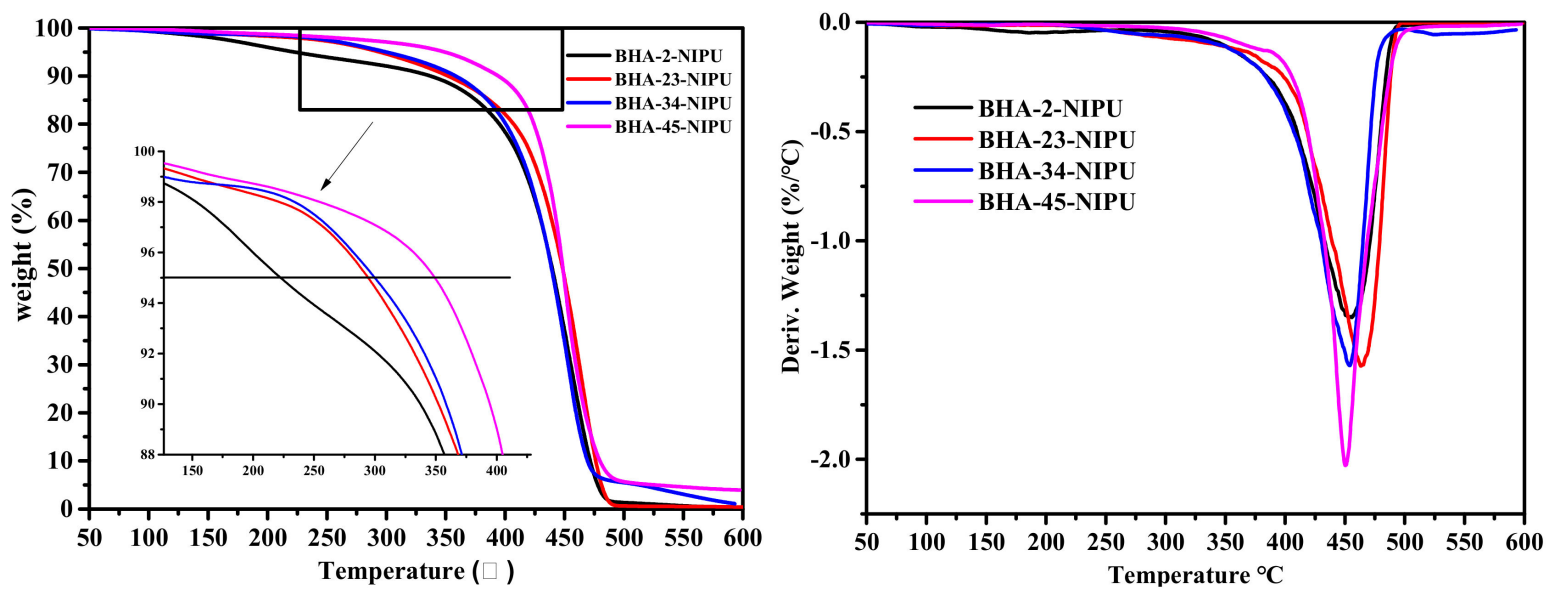
TGA (**left**) and differential thermal gravity (DTG) (**right**) curves of four NIPU-prepolymers.

The thermal stability of NIPU-prepolymers was characterized using TGA, and the results are demonstrated in Figure 5 (left). When the secondary amine number increased, the 5% mass decomposition temperature obviously increased, and the highest temperature was about 375 °C. The root of this result might be because the hydrogen bonds were formed between the different N atoms [39]. Further, according to differential thermal gravity (DTG) curves (Figure 5, right), there was only one maximum decomposition rate (at about 460 °C) in the curve, which illustrated that thermal degradation occurred in one step [40]. Moreover, the maximum decomposition rate slightly decreased as the number of secondary amines increased, and possible explanations may be due to the lower bond dissociation energy of C–N (337.7 kJ/mol) than that of C–C (359.2 kJ/mol) [41].

**Figure 6:**
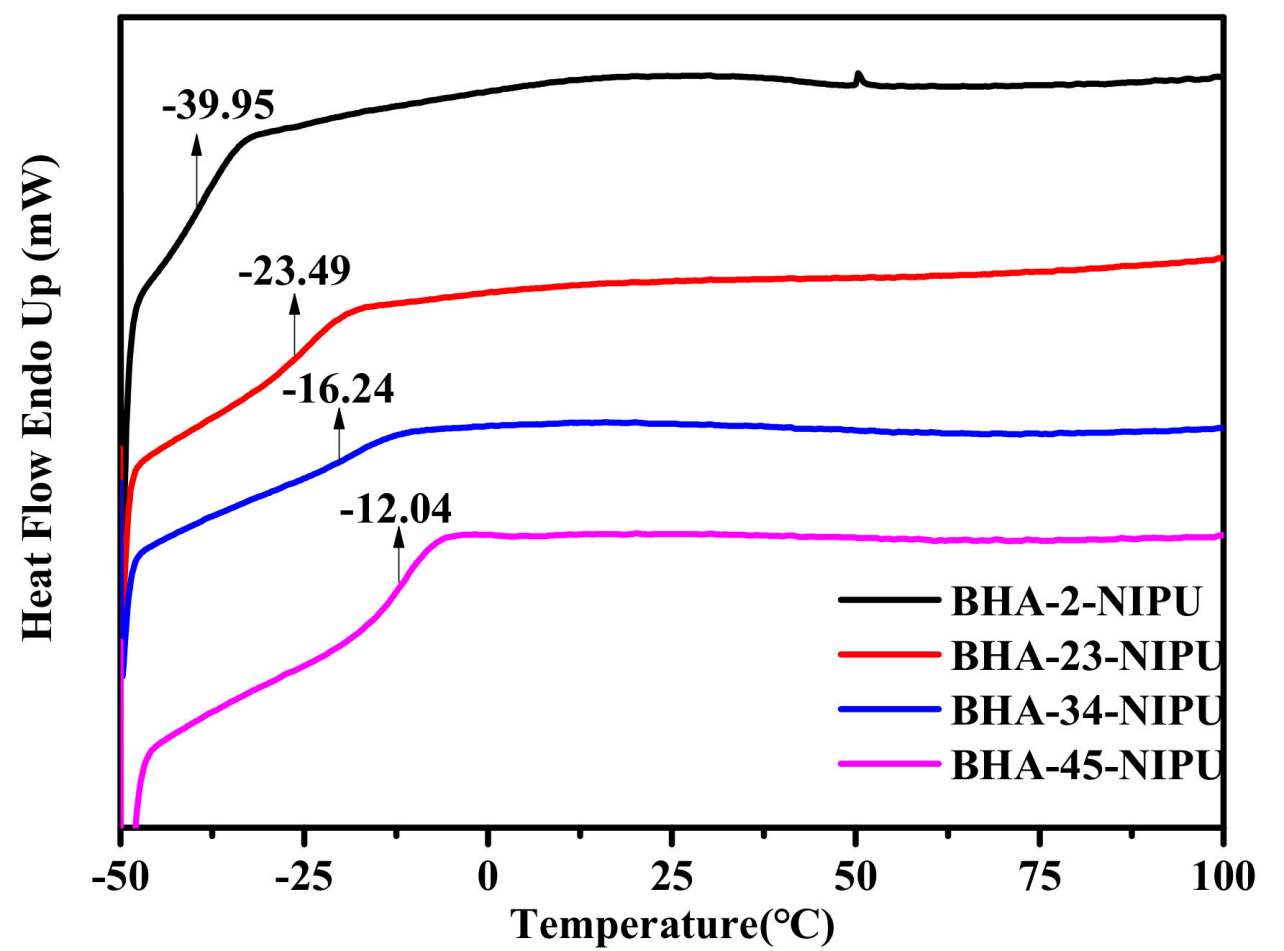
Differential scanning calorimetry (DSC) curves of four NIPU-prepolymer.

The glass transition temperatures of the four NIPU-prepolymers were determined by DSC, and the results were displayed in Figure 6. It can be seen that there was only one *T*_g_ in every DSC curve, indicating that all NIPU-prepolymers were a homogeneous phase system. The results also showed that all *T*_g_ of NIPU-prepolymers were blew zero, and the NIPU-prepolymers formed a viscous flow state, which is ascribed to their low relative molecular weight. In addition, because the significant influence of free hydrogen from the secondary amine number could facilitate the formation of more hydrogen bonds, the *T*_g_ of the NIPU-prepolymers increased as the secondary amine number increased. 

**Figure 7:**
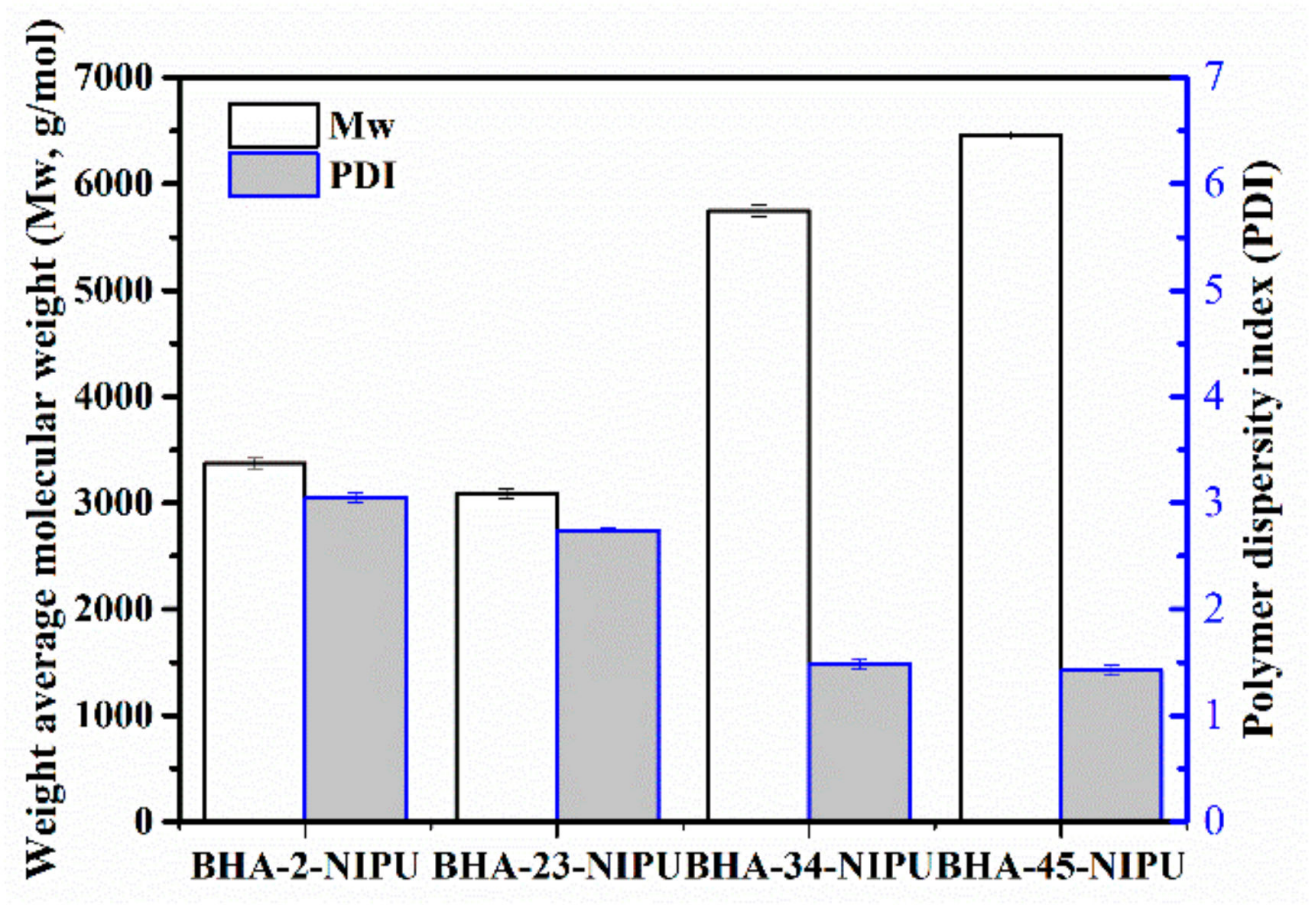
Molecular weight (Mw) and PDI of four NIPU-prepolymers.

Gel permeation chromatography (GPC) was applied to measure the weight average molecular weight (Mw) and polymer dispersity index (PDI), and the results are shown in Figure 7. The Mw of BHA-45-NIPU reached 6460 g/mol, which was the highest. The Mw of BHA-23-NIPU was almost as low as that of BHA-2-NIPU. According to the research, the secondary amine could facilitate polycondensation. One possible reason for this result is that the secondary amine is alkaline and easily reacted with acid as a first process. The trend of PDI also could be explained by the above reason.

### 3.3. Synthesis of HNIPUs

**Figure 8:**
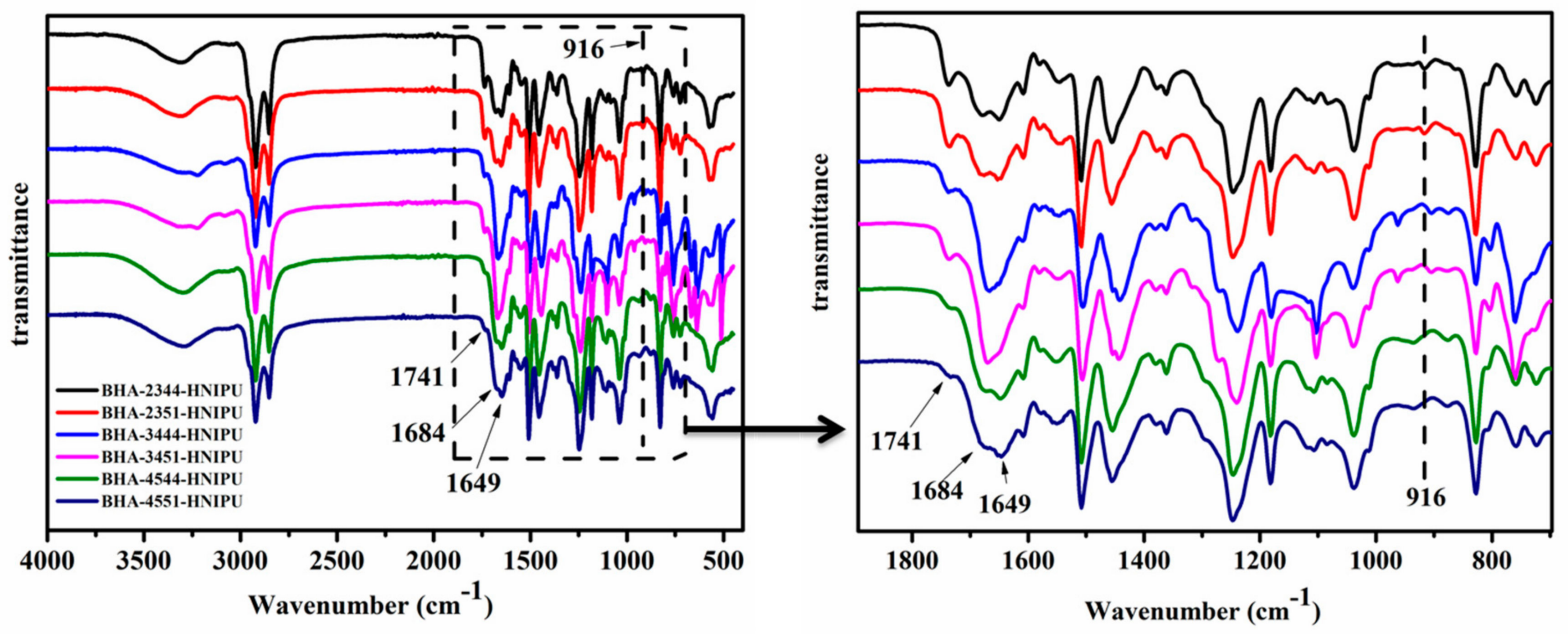
FTIR spectra of HNIPUs.

The main purpose of Figure 8 was to indicate whether the reaction was complete, according to the presence of the peak at 916 cm^−1^ (epoxy group). It can be found that BHA-2344-HNIPU and BHA-2351-HNIPU have residual epoxy groups, mainly because o the low content of secondary amines, which led to lower reaction sites with epoxy groups. In contrast to Figure 4, the band intensity of the vibration peak at 1741 cm^−1^, which belongs to C=O (carboxyl groups), showed an obviously reduction. This result might boil down to the reaction between the carboxyl groups and the epoxy group. The vibration peaks at 1684 cm^−1^ and 1649 cm^−1^ were attributed to the urethane groups and ester groups, respectively. Compared with Figure 4, the C=O vibration peak at 1691 cm^−1^ shifted to lower wavenumbers of 1684 cm^−1^, which indicated that the epoxy group provided additional hydrogen bonding for HNIPU [42,43]. Further, Scheme 4 showed the additional hydroxyl groups because of the ring opening of epoxy groups, which could lead to more hydrogen bonding.

**Figure 9:**
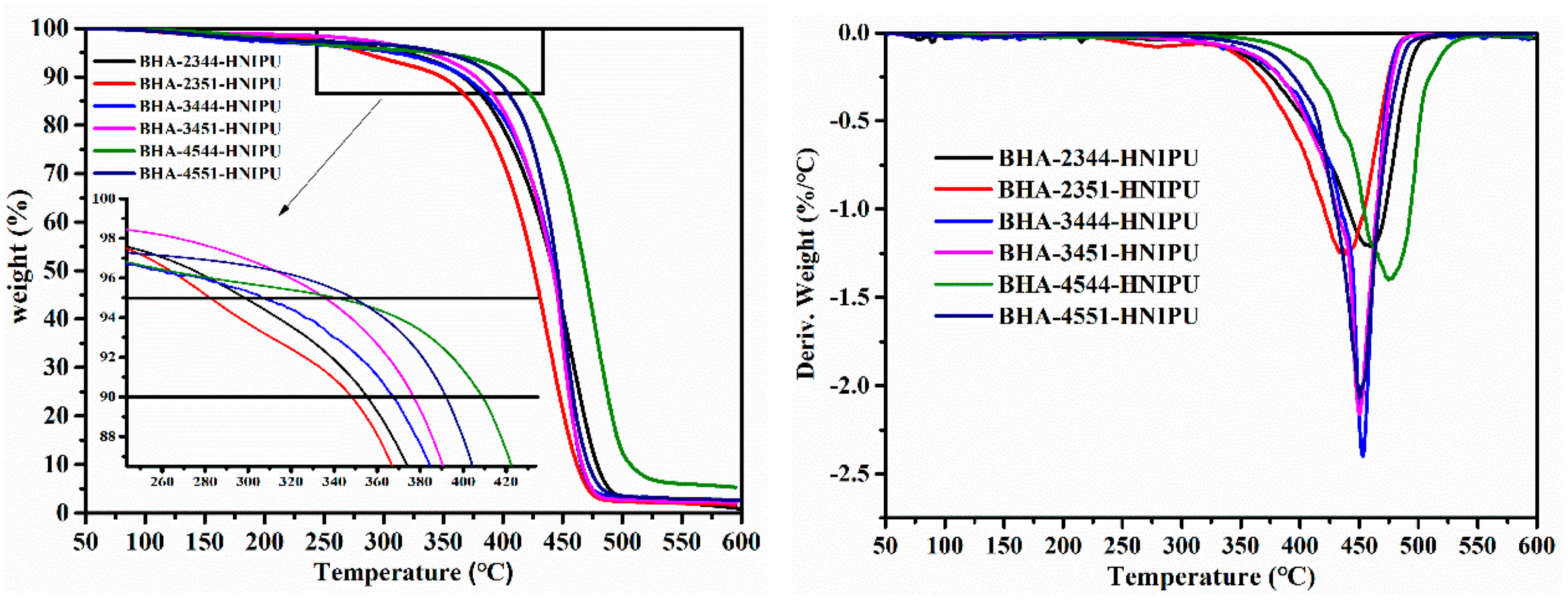
TGA (**left**) and DTG (**right**) curves of HNIPUs.

Figure 9 shows the thermal stability of HNIPUs. According to the partial enlargement of TGA curves, the varied trend affected by secondary amine was consistent with Figure 5, illustrating that thermal stability was mainly affected by the content of the secondary amine. In other words, after reacting with epoxy groups under the condition of the programmed temperature, the chemical bond from the secondary amine still had a good thermal stability and a dominant role. Meanwhile, the E-44 with the lower epoxy value provided better thermal stability than E-51, based on the results of Figure 9 (left). This phenomenon could be explained by the greater number of hydroxyl groups in the structure of E-44, which could influence the hydrogen bond or crosslinking degree. In Figure 9 (right), the BHA-4544-HNIPU clearly had the highest maximum decomposition rate and the BHA-2351-HNIPU had the lowest maximum decomposition rate. 

**Figure 10:**
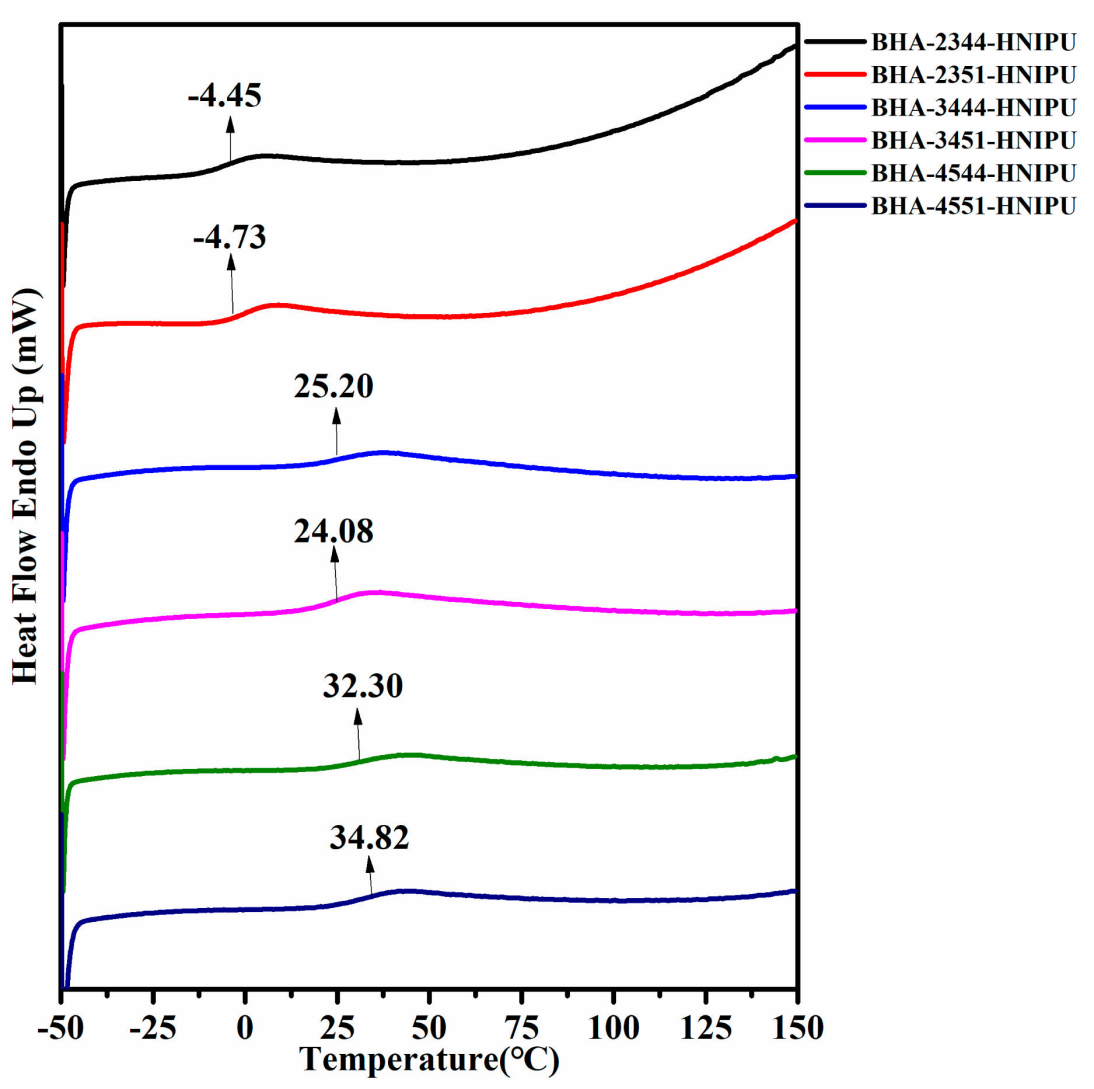
DSC curves of HNIPUs.

As the only secondary amine in BHA-23, the crosslinking degree was naturally low in BHA-2344-HNIPU and BHA-2351-HNIPU. Therefore, these two HNIPUs showed a viscous flow state instead of an elastomeric state at room temperature, and this result was represented in Figure 10. The glass transition temperature (*T*_g_) is affected by many different parameters, such as intermolecular interactions, molecular symmetry, chain stiffness, type of aside group, branching/crosslinking density, and molar mass [44]. In this study, we found that secondary amine content played a bigger role in influencing *T*_g_ than the epoxy value. The BHA-4551-HNIPU had the highest *T*_g_ (34.82 °C) in all HNIPUs. The main reason for this result could be its high crosslinking degree, especially the greater number of sites of crosslinking from the secondary amine.

**Figure 11:**
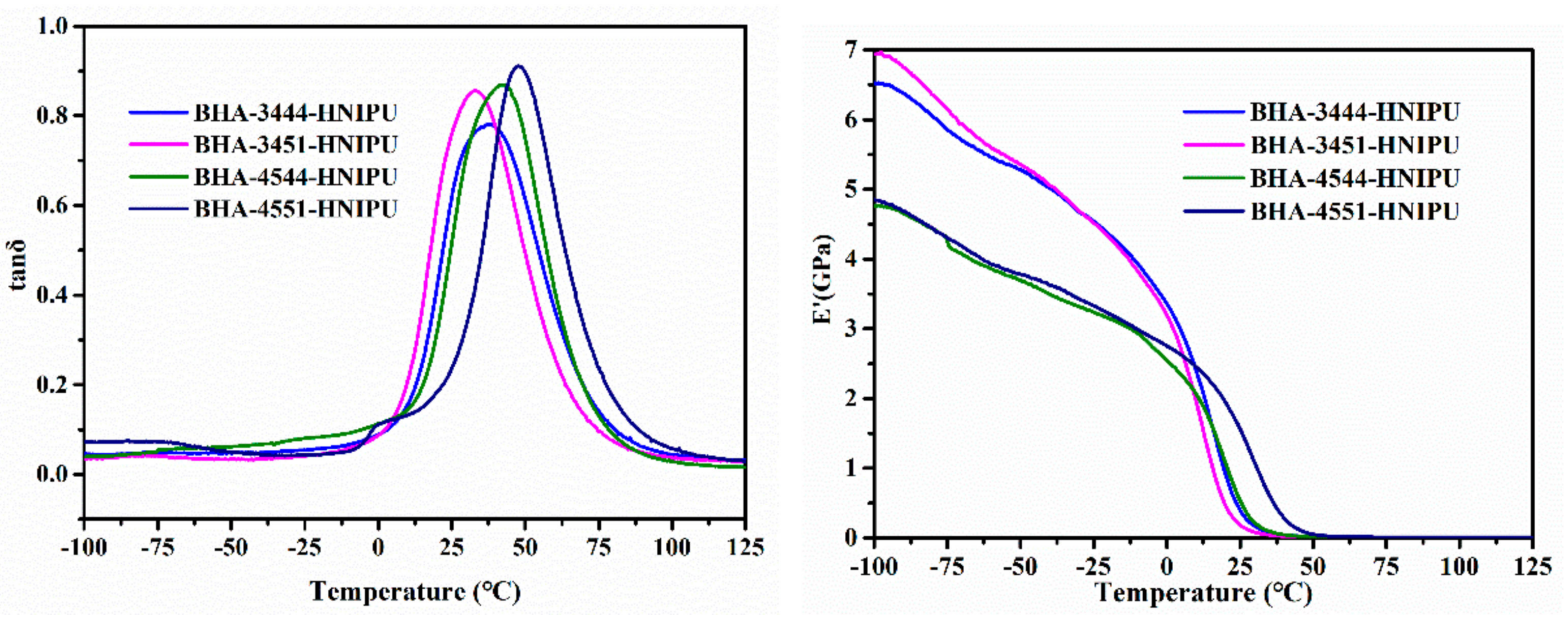
tanδ and E’ of HNIPUs.

Because the DMA tensile mode needs the materials to present an elastomeric state, BHA-2344-HNIPU and BHA-2351-HNIPU, which were a viscous flow state, could not satisfy the testing requirement. Usually, the highest tanδ indicates the *T*_g_, and the results are consistent with that of the DSC (seen in Figure 11). Meanwhile, the regularity of DMA also agrees with the DSC in *T*_g_. The homogeneity of material can be characterized by the tanδ peak: a symmetrical and narrow tanδ reveals a homogeneous material [45]. Consequently, a loss factor tanδ shows that HNIPUs are homogeneous. 

The storage modulus E’ was used to illustrate the rigidity of material. The storage modulus of BHA-3444-HNIPU and BHA-3451-HNIPU were higher than those of BHA-4544-HNIPU and BHA-4551-HNIPU before 13 °C, which could be attributed to the crystallization behavior of these two HNIPUs. However, BHA-4551-HNIPU had the highest storage modulus after 13 °C, and the other three HNIPUs just had a tiny difference. This result shows that the BHA-4551-HNIPU had the best rigidity of all HNIPUs after 13 °C, and demonstrates that the crosslinking degree was important for the storage modulus.

Crosslinking density (*ν_e_*) was calculated by the following equation:

(3) $$\nu_{e}=\frac{E\prime_{at\;T\alpha +50}}{3RT_{\alpha +50}}$$

where E’_at Tα+50_ is the storage modulus at the rubbery region, R is the gas constant, and T_α+50_ (K) is the temperature in the rubbery region (of transition from the vitreous to elastic domain of material determined at the maximum of the tanα curve [45,46]. According to Table 4, the BHA-4551-HNIPU had the highest crosslinking density of all HNIPUs, which was in accordance with the results of TGA and DSC. 

**Figure 12:**
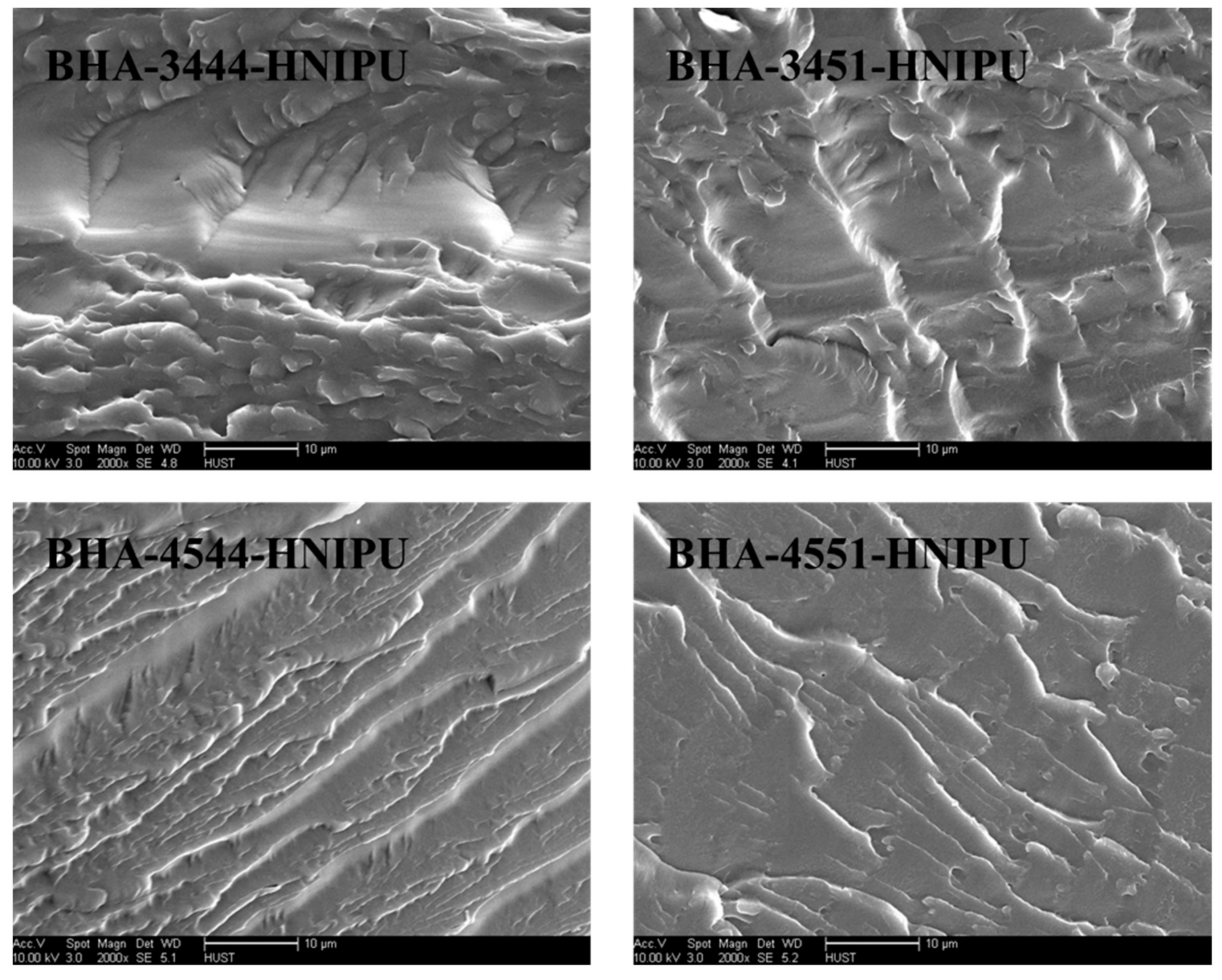
SEM images of HNIPUs.

The Figure 12 showed surface morphology of the four HNIPUs. It was evident that the materials had a layer structure in Figure 12 of BHA-3444-HNIPU, BHA-3451-HNIPU and BHA-4544-HNIPU. In contrast, BHA-4551-HNIPU had a more continuous surface, and the others had an obviously stratified structure, which confirmed that BHA-4551-HNIPU had a better rigidity than the other HNIPUs. This result was consistent with the DMA test. Moreover, this could make it possible for this material to be applied in coating.

**Figure 13:**
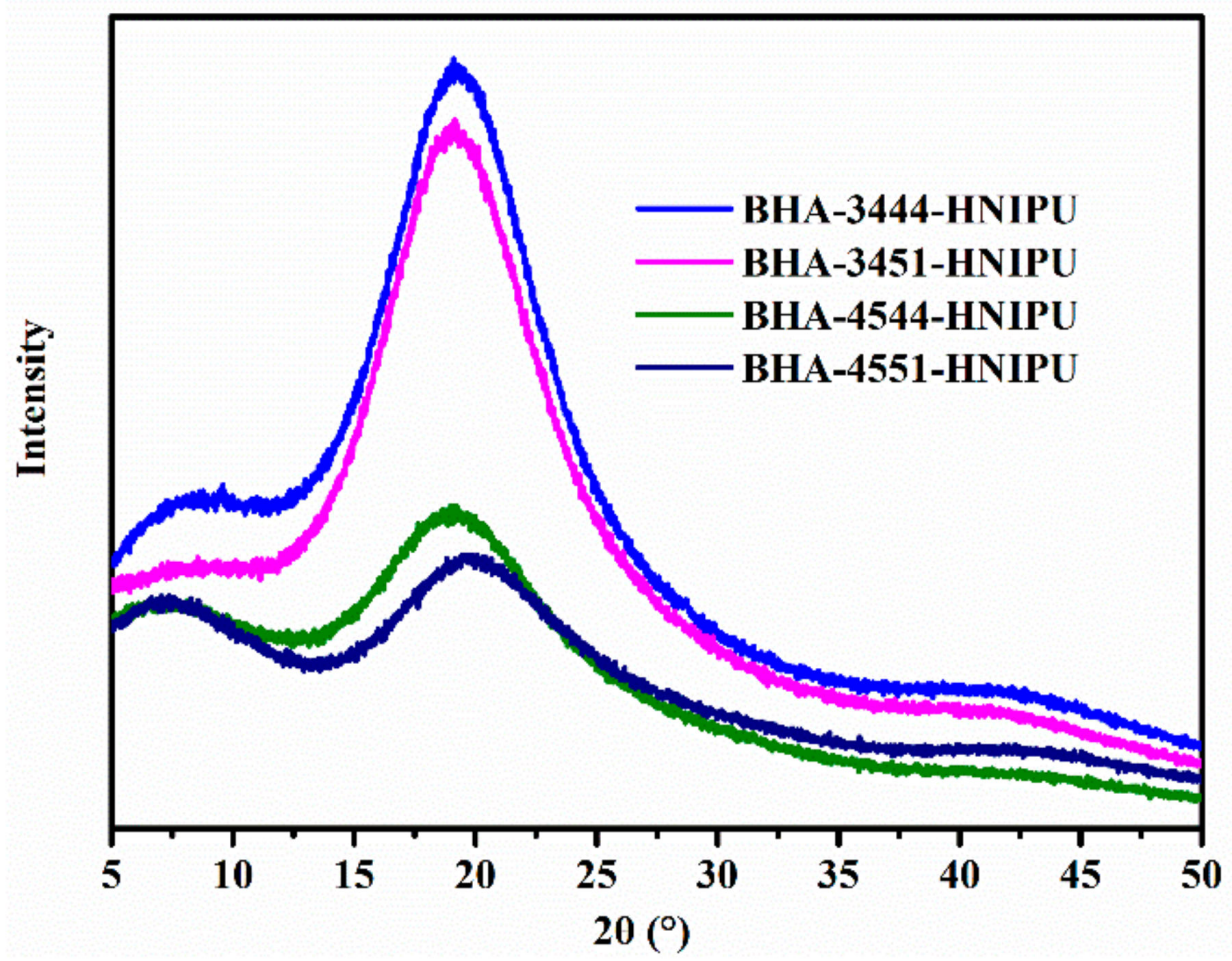
XRD pattern of HNIPUs.

In order to understand the crystallization behavior of the HNIPUs, XRD analysis was necessarily investigated. As seen in Figure 13, there were no prominent diffraction peaks, and this result indicates that BHA-4544-HNIPU and BHA-4551-HNIPU were structurally amorphous. However, BHA-3444-HNIPU and BHA-3451-HNIPU might form crystallization, which could be explained by the lower secondary amine content and lower crosslinking degree. Therefore, the crystallization behavior of HNIPUs could be tailored based on the secondary content.

**Figure 14:**
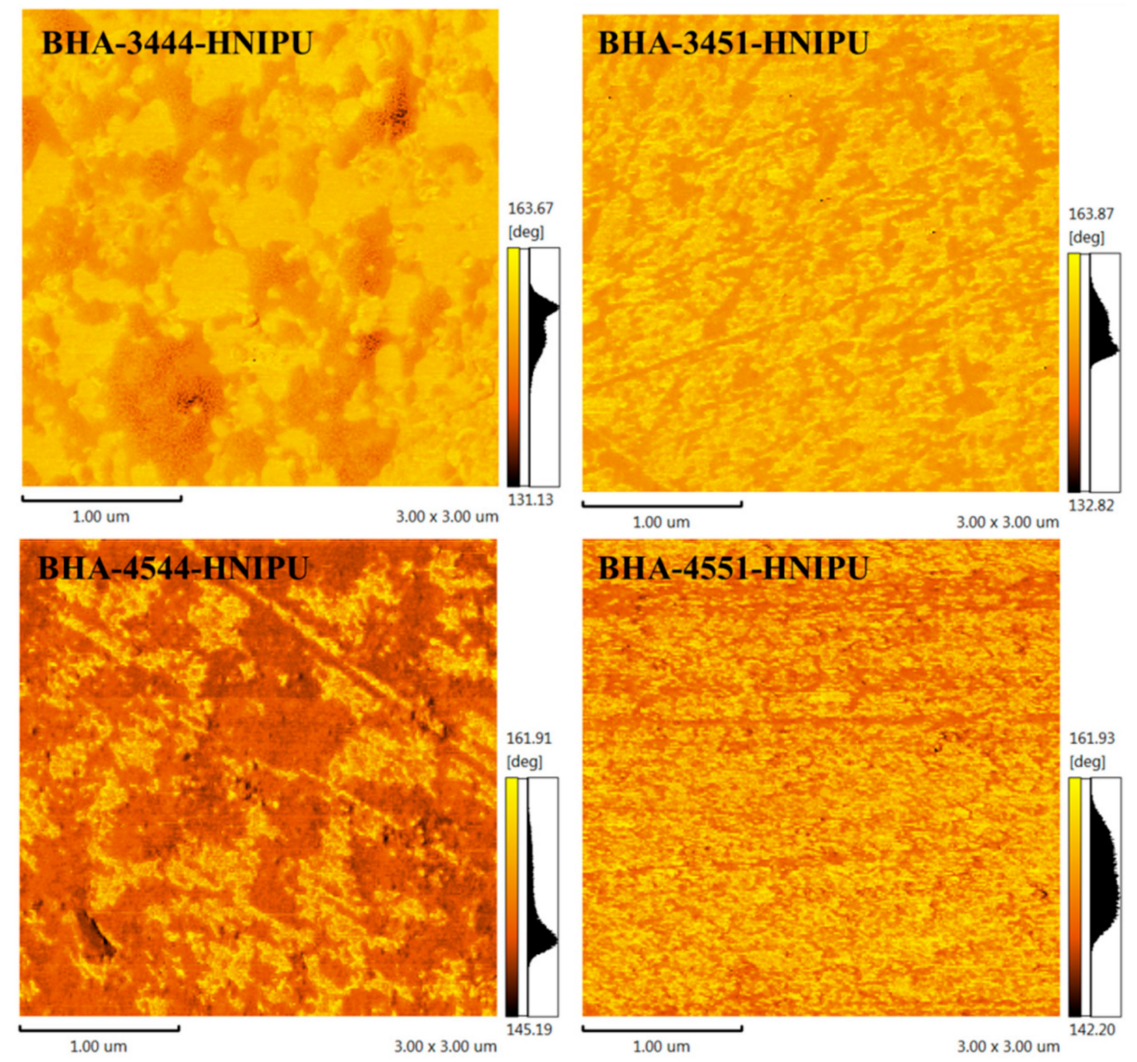
Surface morphology of HNIPUs characterized by AFM phase imaging.

AFM was an appropriate morphological surface characterization method for studying the nanophase separation of HNIPUs. As shown in Figure 14 of BHA-3444-HNIPU, BHA-3451-HNIPU and BHA-4544-HNIPU, the two phases (the bright domain represents the soft segment and the dark domain represents the hard segment) were apparently heterogeneous, especially for BHA-3444-HNIPU and BHA-4544-HNIPU; in other words, the E-44 had a larger impact on nanophase separation than E-51. Furthermore, amine type played a less important role than epoxy value because it could only determine the hard segment, and BHA-4551-HNIPU naturally had the best microphase separation (seen in Figure 14 of BHA-4551-HNIPU). For this reason, BHA-4551-HNIPU showed wonderful thermodynamic properties as a result of the TGA and DMA shown.

**Figure 15:**
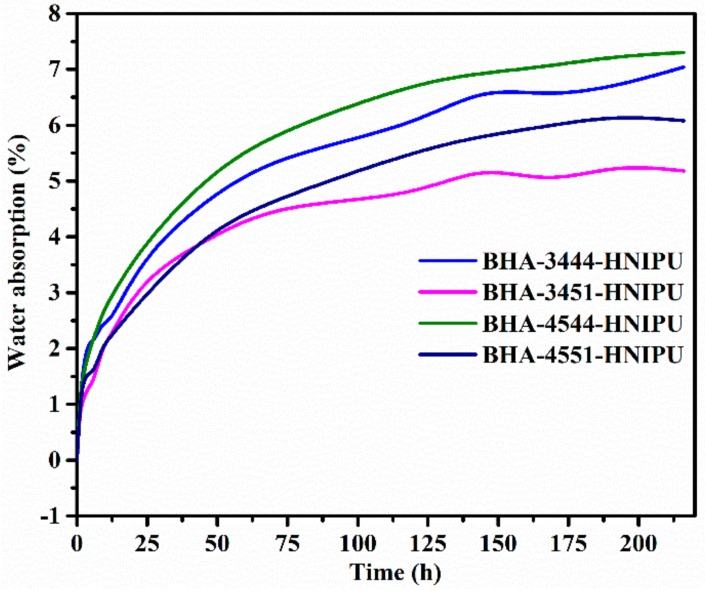
Water absorption of HNIPUs.

The water absorption of HNIPUs was studied for water tolerance in Figure 15. After 125 h, water absorption of the materials began to stabilize. Theoretically, the higher crosslinking degree could lead to worse water absorption [47], but BHA-3451-HNIPU had the worst water absorption of all HNIPUs. By contrast, E51 could bring a higher epoxy value than E44 and made a higher crosslinking degree. This did lead to a worse water absorption. However, a higher secondary amine could make the HNIPU reserve more water via a hydrogen bond. This hydrogen bond could have a more important role in water absorption than the influence of the epoxy value, so the result showed that BHA-3451-HNIPU had the best water tolerance among all HNIPUs.

**Figure 16:**
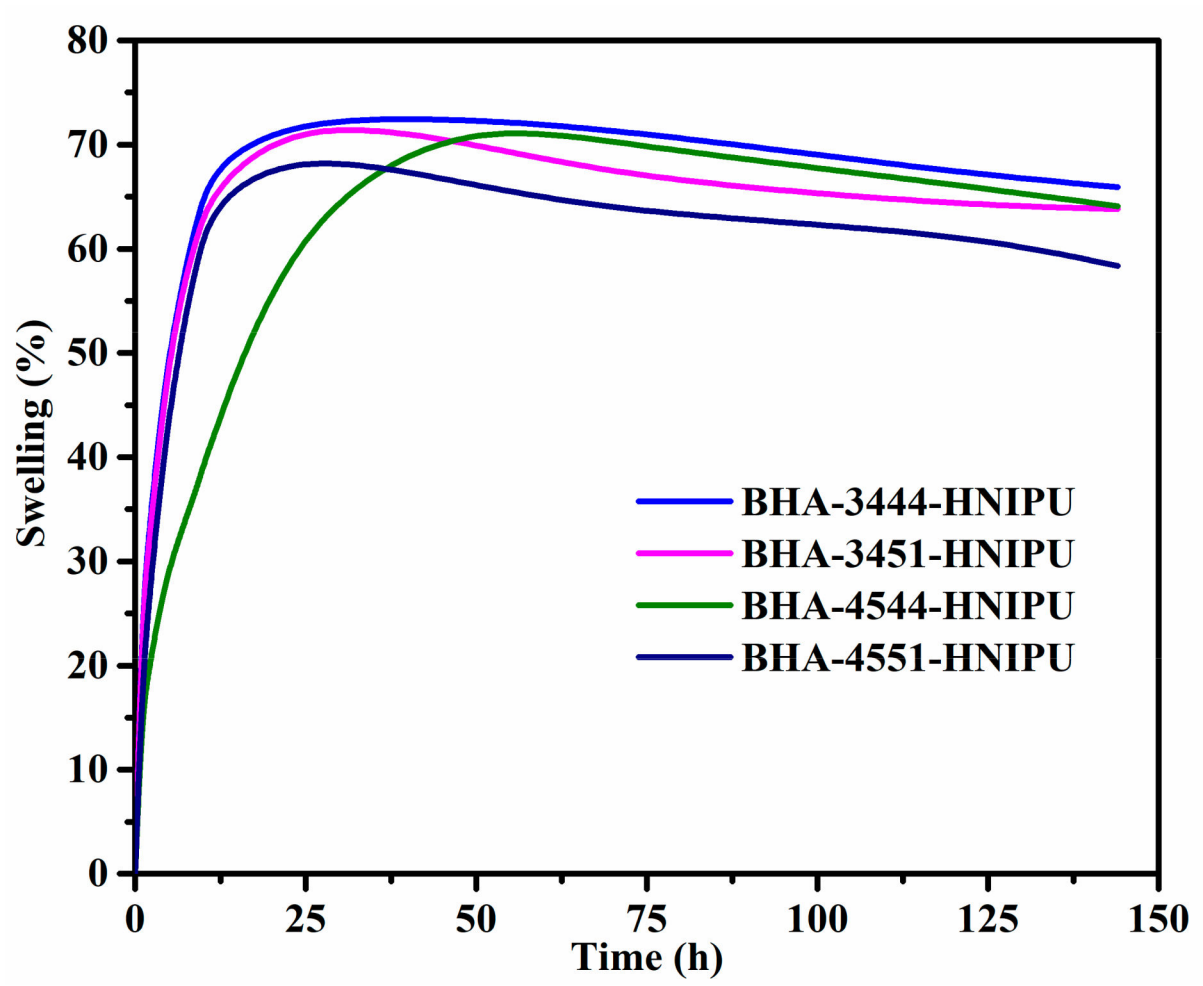
Swelling of HNIPUs.

Swelling was also an instruction of the crosslinking degree for HNIPUs. In this study, DMSO was chosen as a solvent, which cannot dissolve these polymers and just makes them swell. As Figure 16 shows, BHA-4551-HNIPU had the greatest swelling in these four HNIPUs, which indicates that it has the highest crosslinking degree. Meanwhile, the epoxy value was a more critical influence than the secondary amine content on swelling. In other words, the epoxy value had a noticeable impact on crosslinking degree instead of secondary amine content. The greatest swelling of the HNIPUs was less than 70%, which suggests that its environmental stabilities might be reasonably good.

## 4. Conclusions

In this study, four BHAs were synthesized as reactants for an NIPU-prepolymer. Then, epoxy resin was selected as the crosslinking agent of NIPU via an environment friendly approach. Accordingly, with crosslinking with epoxy groups, the properties of HNIPUs had been greatly improved on *T*_g_ and thermal stability. A series of tests had confirmed the correct chemical constructions of the synthesized products and diverse properties of those samples. Among them, BHA-4551-HNIPU had the highest crosslinking degree in all synthesized HNIPUs, which led to the highest thermal stability with mutually confirmed characterization via TGA, DMA, SEM, and AFM. According to these results, this HNIPU possesses the application potential of heat resistant coatings. Moreover, it was found that the regularity of the crosslinking degree, epoxy value, and secondary amine: epoxy value played a more important role for crosslinking degree than secondary amine content. Meanwhile, it provided an ingenious method to synthesize HNIPU via renewable materials for the polyurethane industry.

## Figures and Tables

**Table 1:** The raw materials mass and results of the BHAs. EDA, ethanediamine; DETA, diethylenetriamine; TETA, trethylenetriamine; TEPA, diethylenetriamine; EC, Ethylene carbonate.

BHA Type	Amine Type	Mole Ratio (Amine:EC)	Mole Ratio	EC Mass	Amine Mass	BHA Mass	Yield
BHA-2	EDA	1:2	1:1	100.0 g	34.1 g	127.4 g	95%
BHA-23	DETA	1:2	1:1	100.0 g	58.0 g	148.5 g	94%
BHA-34	TETA	1:2	1:1	100.0 g	82.2 g	174.9 g	96%
BHA-45	TEPA	1:2	1:1	100.0 g	118.2 g	207.3 g	95%

**Table 2:** The raw material mass and results of the NIPUs.

NIPU Type	BHA Type	Mole Ratio (BHA:DA)	BHA Mass	DA Mass	NIPU Mass	Yield
NIPU-2	BHA-2	1:1.05	10.0 g	38.9 g	43.3 g	89%
NIPU-23	BHA-23	1:1.05	10.0 g	28.0 g	32.9 g	87%
NIPU-34	BHA-34	1:1.05	10.0 g	17.9 g	25.0 g	90%
NIPU-45	BHA-45	1:1.05	10.0 g	15.7 g	21.8 g	85%

**Table 3:** The raw materials mass and results of HNIPUs.

HNIPU Type	NIPU Type	EP Type	Mass Ratio (NIPU:EP)	NIPU Mass	EP Mass	HNIPU Mass	Yield
HNIPU-2344	NIPU-23	E44	1:0.7	2.0 g	1.4 g	3.4 g	100%
HNIPU-2351	NIPU-23	E51	1:0.7	2.0 g	1.4 g	3.4 g	100%
HNIPU-3444	NIPU-34	E44	1:0.7	2.0 g	1.4 g	3.4 g	100%
HNIPU-3451	NIPU-34	E51	1:0.7	2.0 g	1.4 g	3.4 g	100%
HNIPU-4544	NIPU-45	E44	1:0.7	2.0 g	1.4 g	3.4 g	100%
HNIPU-4551	NIPU-45	E51	1:0.7	2.0 g	1.4 g	3.4 g	100%

**Table 4:** HNIPU parameters obtained from the DMA.

Sample	T_α_ (°C)	T_α_ + 50 (°C)	E’ _at Tα+50_ (MPa)	ν_e_ (mol/m^3^)
BHA-3444-HNIPU	38.04	88.04	7.95	881.30
BHA-3451-HNIPU	32.87	82.87	7.29	821.74
BHA-4544-HNIPU	42.32	92.32	6.35	696.34
BHA-4551-HNIPU	47.50	97.50	8.56	925.33

## Supplementary Materials

The following are available online at https://www.mdpi.com/2073-4360/11/6/1026/s1, Figure S1: FTMS spectrum of BHA-2, Figure S2: FTMS spectrum of BHA-23, Figure S3: FTMS spectrum of BHA-34, and Figure S4: FTMS spectrum of BHA-45
