# Supplementary material for: Solvent- and Catalyst-free Synthesis, Hybridization and Characterization of Biobased Nonisocyanate Polyurethane (NIPU)
Source: Polymers (Basel). 2019 Jun 10;11(6):1026. doi: 10.3390/polym11061026 (PMC6631551; doi:10.3390/polym11061026)
Supplement: Supplementary file 1 [file polymers-11-01026-s001.pdf]

# Supplementary Material

## Solvent- and catalyst-free synthesis, hybridization and characterization of biobased nonisocyanate polyurethane (NIPU)

**Xin He, Xiaoling Xu, Qian Wan, Guangxu Bo and Yunjun Yan** *

Key Laboratory of Molecular Biophysics of the Ministry of Education, College of Life Science and Technology, Huazhong University of Science and Technology, Wuhan 430074, China

Experimental section

**Figure S1:** FTMS spectrum of BHA-2

**Figure S2:** FTMS spectrum of BHA-23

**Figure S3:** FTMS spectrum of BHA-34

**Figure S4:** FTMS spectrum of BHA-45

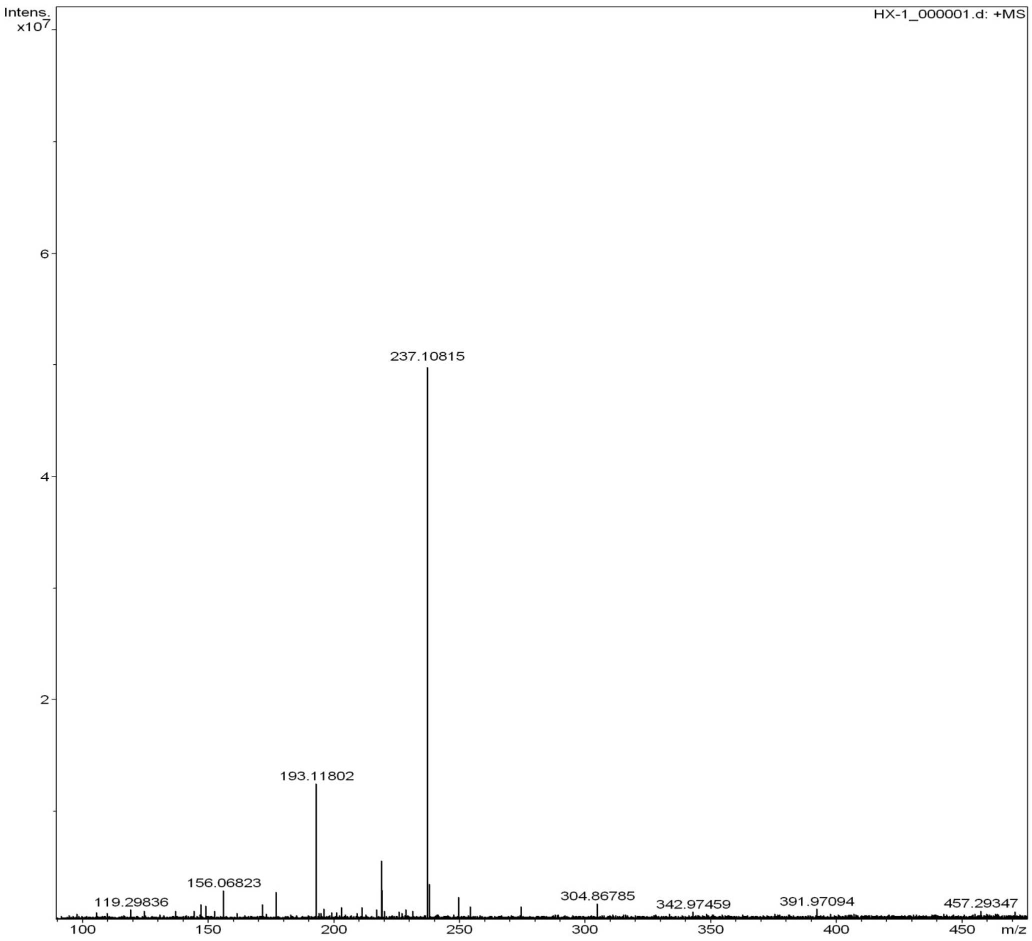


**Figure 1S.** FTMS spectrum of BHA-2

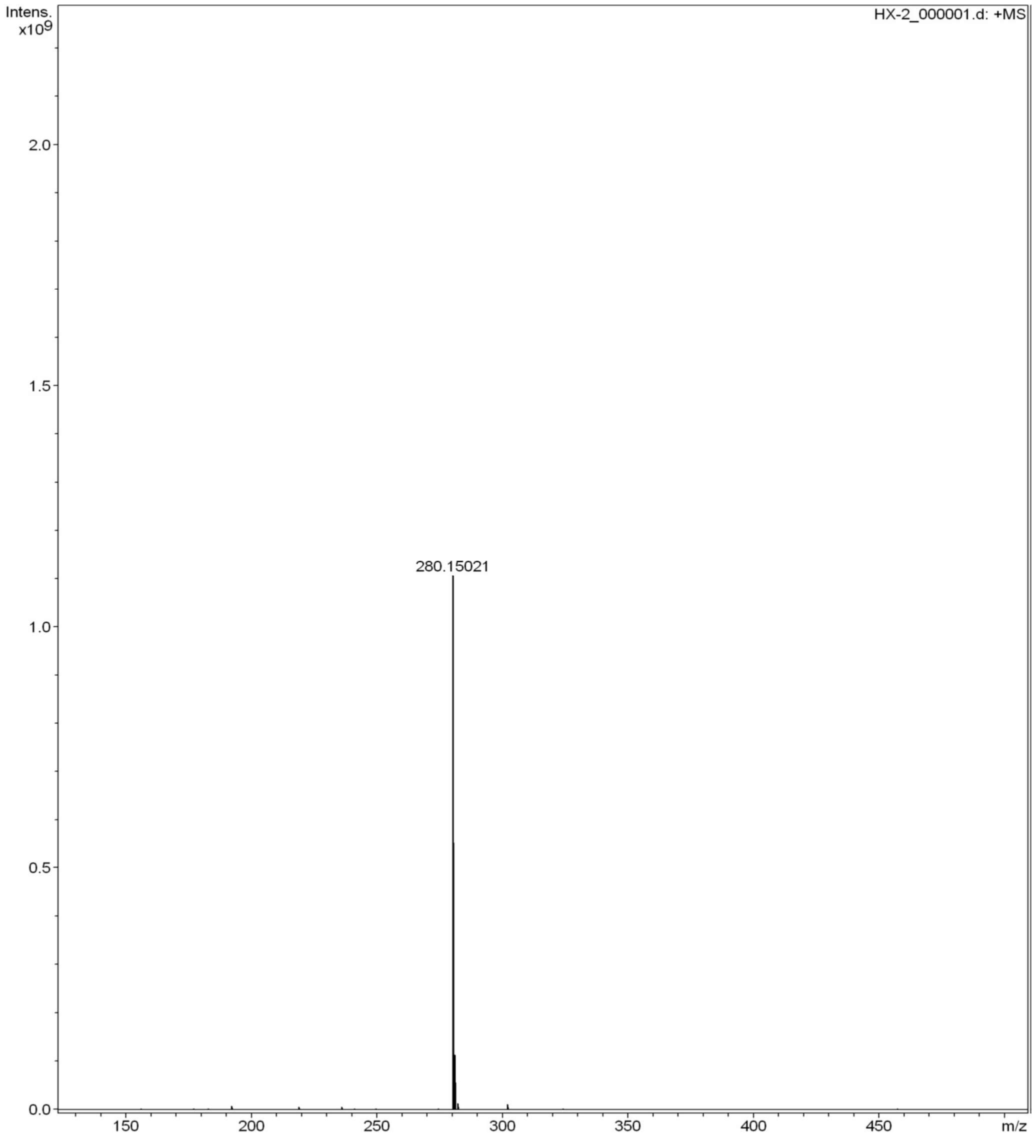


**Figure S2:** FTMS spectrum of BHA-23

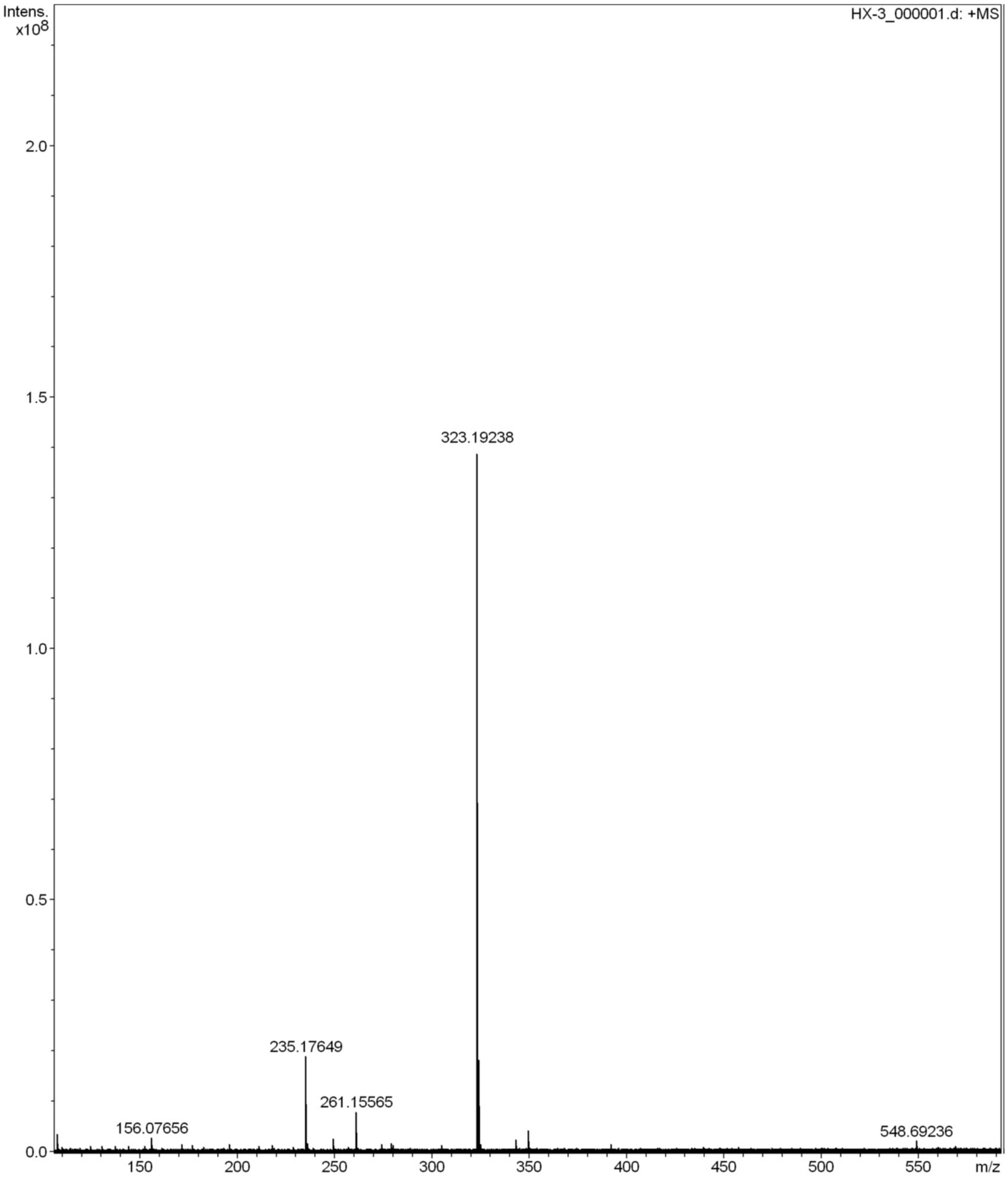


**Figure S3:** FTMS spectrum of BHA-34

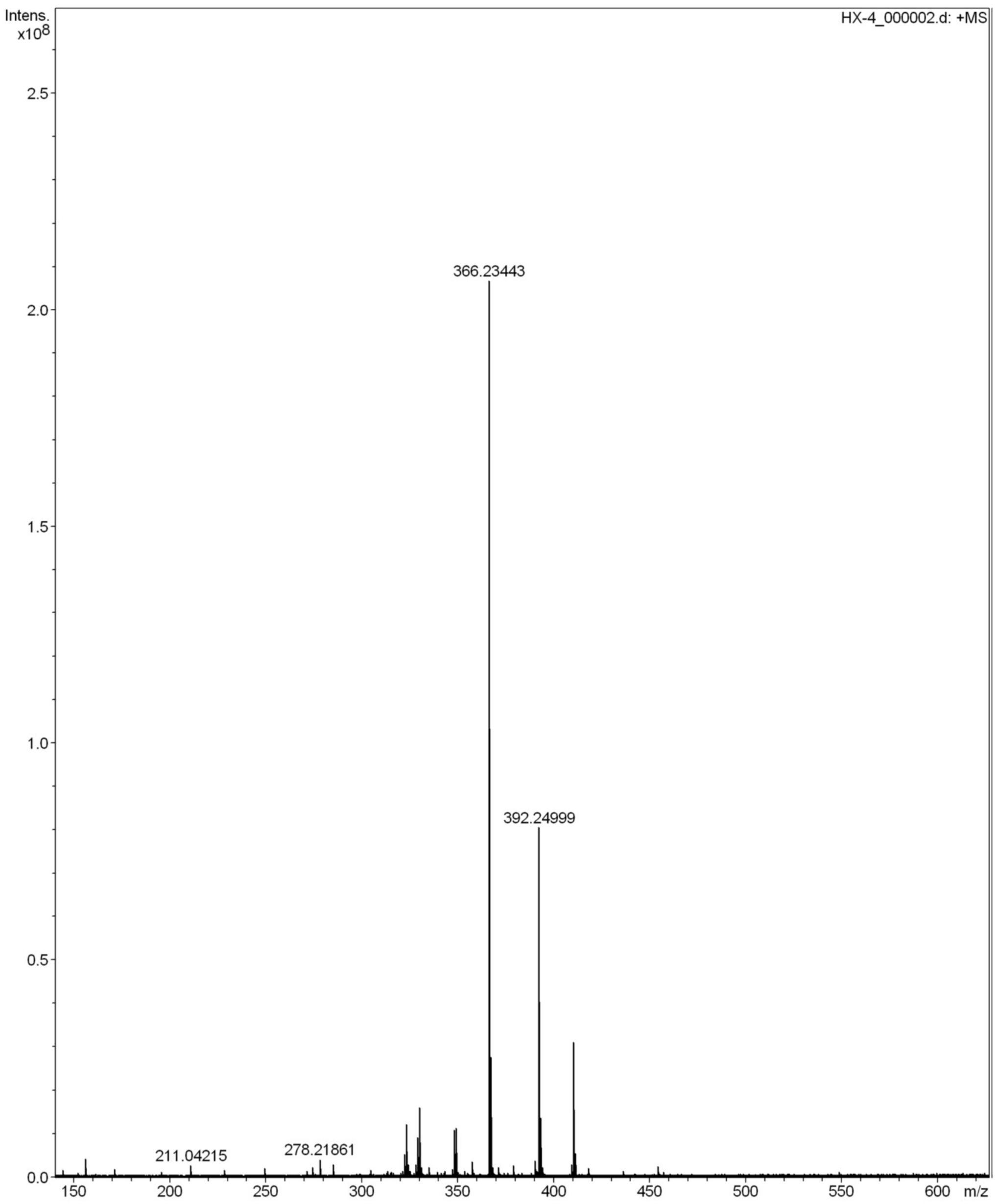


**Figure S4:** FTMS spectrum of BHA-45
